# 2’-FL and cross-feeding bifidobacteria reshaped the gut microbiota of infants with atopic dermatitis ex vivo and prevented dermatitis in mice post-microbiota transplantation through retinol metabolism activation

**DOI:** 10.1080/19490976.2025.2474148

**Published:** 2025-03-02

**Authors:** Ce Qi, Zhongxia Li, Huayu Tu, Fang Sun, Wenbo Guo, Can Di, Ruikun He, Xiaolei Ze, Lintao Zhang, Ruijuan Gao, Pengyue Hu, Wenjing Yang, Kexin Li, Jiayi Liu, Xiaonan Pan, Zilu Jin, Jin Sun

**Affiliations:** a Institute of Nutrition and Health, Qingdao University, Qingdao, China; b BYHEALTH Institute of Nutrition & Health, Guangzhou, China; c Pediatrics, Jiaozhou Maternal and Child Health and Family Planning Service Center, Qingdao, China

**Keywords:** 2’-fucosyllactose, bifidobacteria, atopic dermatitis, retinol metabolism, cross-feeding

## Abstract

2’-Fucosyllactose (2’−FL), a predominant human milk oligosaccharide, plays a crucial role in the development of the infant gut microbiota and immune system. However, the microbiota of infants with atopic dermatitis (AD) often has difficulty utilizing 2’-FL. Here, we found that strains from human milk, *Bifidobacterium bifidum* FN120 and *Bifidobacterium longum* subsp. *longum* FN103, utilized 2’-FL for growth by cross-feeding. Through an *ex vivo* continuous fermentation system, we found that 2’-FL and cross-feeding bifidobacteria synergistically enhanced the production of short-chain fatty acids (SCFAs), particularly acetate and propionate, while reshaping the gut microbiota in infants with AD. The reshaped microbiota was then transplanted into oxazolone-induced mice. We observed that AD symptoms in mice were effectively prevented, with significant changes in the ileum microbiota and increased intestinal SCFA levels. RNA sequencing analysis of Peyer’s patches in the small intestine revealed activation of the retinol metabolic pathway. Nontargeted metabolomics analysis revealed a significant increase in plasma retinoate levels, which correlated markedly with AD-related markers. Collectively, our study demonstrated that supplementation with cross-feeding bifidobacteria and 2’-FL reshaped the gut microbiota, activated retinol metabolic pathways, promoted immune tolerance, and thereby prevented AD. Our findings provide novel insights into the therapeutic potential of combining prebiotics and probiotics to modulate the gut – skin axis and support immune tolerance in early life, offering a promising strategy for infantile AD management and prevention.

## GRAPHICAL SUMMARY

*Bifidobacterium bifidum* FN120 and*Bifidobacterium longum* subsp. *longum* FN103, derived from breast milk, synergistically utilize 2’-FL, reshaped the gut microbiota and enhanced short-chain fatty acid production in an in vitro fermentation model of AD infants. Transplantation of this microbiota into mice with oxazolone-induced AD alleviated symptoms by activating retinoid metabolism.

## GRAPHICAL ABSTRACT

**Figure:**
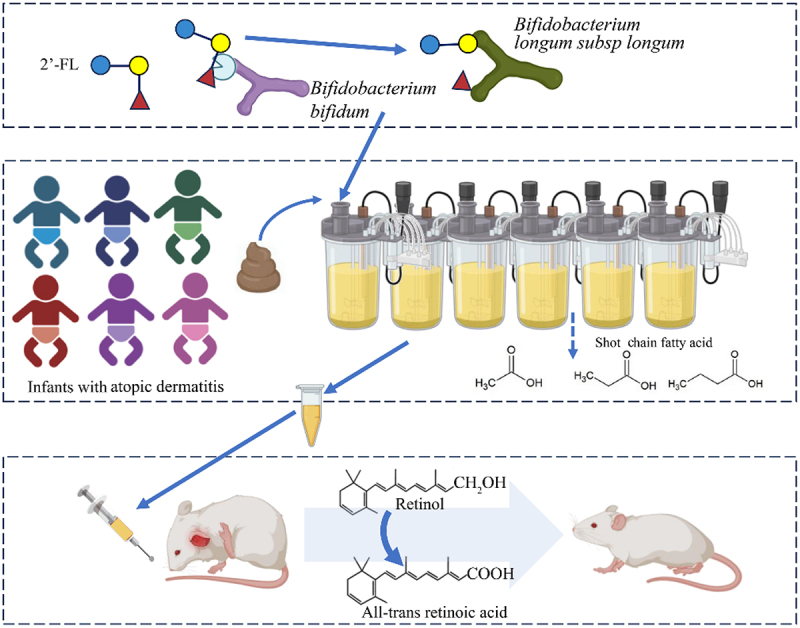

## 1. Introduction

Human milk oligosaccharides (HMOs) promote a healthy gut microbiota and exhibit protective effects against atopic dermatitis (AD).^1^ The prevalence of AD has increased alongside the rise of industrialization and urbanization.^2^ This condition, characterized by an exaggerated immune reaction leading to the production of immunoglobulin E (IgE) in response to external stimuli,^3^ is the most common atopic disorder in children and manifests as red, scaly, and itchy skin.^4^ Healthy immune system development is crucial in modulating AD, and emerging evidence highlights the immunomodulatory functions of the gut microbiome. Specifically, the occurrence of AD is associated with low diversity of the intestinal microbiota in early life, a high proportion of pathogens such as *Klebsiella* and *Escherichia coli*, and a depletion of beneficial bacteria such as *Bifidobacterium* and *Bacteroides*.^5,6^ Despite these associations, the literature presents conflicting findings regarding the protective influence of breastfeeding on AD. Some studies suggest a beneficial effect, whereas others indicate no significant reduction in hereditary or age-specific AD incidence.^7^ This variability in the protective effect of human milk against AD may be attributed to the heterogeneity in the gut microbiota composition of infants and their respective capacities to metabolize HMOs.^8^

Human milk significantly influences the early development of the infant gut microbiota, playing a pivotal role in modulating the maturation of both the innate and adaptive mucosal immune systems.^9^ Given the crucial role of the gut microbiome in the development and progression of AD, modulating it through prebiotics and probiotics may represent a promising therapeutic approach. The levels of 2’-fucosyllactose (2’−FL), a prevalent HMO, correlate with a higher prevalence of nonallergic and nonsensitized infants.^10^ Oral administration of 2’-FL reduces allergic sensitization by promoting the production of short-chain fatty acids (SCFAs) by the gut microbiota.^11^ However, in children with AD, the dysregulated intestinal microbiota has a lower capacity to ferment 2’-FL to produce SCFAs, which may alleviate AD symptoms.^12^ 2’-FL has the capacity to regulate the microbiota of individuals with dysbiosis.^13^ Healthy infants present a typical infant-type microbiome with high levels of *Bifidobacterium bifidum*, whereas allergic infants present an adult-type microbiome with high levels of *Bifidobacterium adolescentis*.^14^ *B. bifidum* is instrumental in establishing HMO-degrading properties in the gut of breastfed infants. As an extracellular degrader of HMOs, *B. bifidum* stimulates the growth of other symbiotic species by releasing products of HMO degradation as cross-feeding substrates, such as galactose, fucose, and lactose.^15^ The cooperation between bifidobacterial strains may increase the ability of the microbiota to degrade HMOs and facilitate the shift of bacterial communities toward those observed in a healthy infant gut microbiome.^13^ *B. bifidum* has been shown to increase the utilization of 2’-FL by other bacteria in chemostat cultures.^16^ However, the extent to which this interaction can effectively modulate the intestinal microbiota in infants with complex AD requires further investigation. It is hypothesized that the supplementation of bifidobacteria capable of cross-feeding and metabolizing 2’-FL may restructure the intestinal microbiota in infants with AD, thereby potentially regulating the immune balance to facilitate the improvement of AD.

This study aims to assess the impact of two *Bifidobacterium* strains isolated from human milk, *B. bifidum* FN120 and *Bifidobacterium longum* subsp. *longum* FN103, which are capable of cross-feeding and metabolizing 2’-FL, on the intestinal microbiota of infants with AD within an *in vitro* continuous fermentation system. Furthermore, the reshaped microbiota was transplanted into mice to investigate its preventive effects against oxazolone (OXA)-induced AD.

## 2. Materials and methods

### 2.1. Single and coculture growth in the presence of 2’-FL

*B. bifidum* FN120 and *B. longum* subsp. *longum* FN103, which were previously isolated from human milk in Tongwei, a county with low AD incidence in China, are stored at the Institute of Nutrition and Health, Qingdao University. The complete annotated genome sequence of FN120 and FN103 have been deposited in the GenBank database under the BioProject ID of PRJNA1218428 and PRJNA1218424, respectively. The phylogenomic tree (Figure S1) based on 92 single-copy gene sequences showed that FN120 and FN103 formed a distinct phylogenetic lineage, against the same strain with complete genome sequence available on NCBI database, respectively. The cultures were subsequently transferred into de Man Rogosa and Sharpe (MRS) broth three times for revival and inoculated into modified MRS broth medium in an anaerobic chamber (COY Laboratories, USA), at which time sucrose was replaced with 1% 2’-FL (Carbosynth Ltd., Berkshire, UK) at a 5% inoculum concentration. They were either cultured individually or cocultured at a 1:1 ratio under anaerobic conditions at 37°C. The optical density (OD_600_) was assessed every 6 hours over a 48-hour period, with each experiment being conducted in triplicate.

### 2.2. Donor recruitment and feces collection in infants with AD

The study was approved and supervised by the Ethics Committee of Qingdao University Medical School (No. QDU-HEC-2022224), ensuring that all investigations were conducted with strict adherence to ethical protocols. A total of six fecal samples from six male exclusively breastfed infants with AD were collected upon request. Only male infants were included in the study to avoid the interference of sex-related intestinal microbiota differences^17^ on the intervention results when the sample size was small. All donors who were diagnosed with AD according to the Williams criteria,^18^ were approximately 37 days old and had not been exposed to antibiotics or probiotics (Table S1). Informed consent was obtained from the mothers of these infants with AD. Feces from an age-matched healthy male infant were also collected for use as a control in subsequent microbiota transplantation experiments. The collected fecal samples were placed in vacuum-sealed bags equipped with oxygen absorbers and kept at low temperatures for transportation to the laboratory. Upon arrival, the feces were homogenized in a sterile stomacher homogenizer (LC-08, Ningbo Licheng Instrument Co., Ltd., Ningbo, China) under anaerobic conditions to prepare fecal bacterial suspensions. These suspensions were subsequently centrifuged at 1000 rpm for 10 minutes, after which the supernatants were transferred into sterile anaerobic tubes. The samples were then stored at −80°C with the addition of 20% glycerol for subsequent *in vitro* fermentation experiments.

### 2.3. Continuous colon microbiota fermentation and treatments

**Figure 1.**
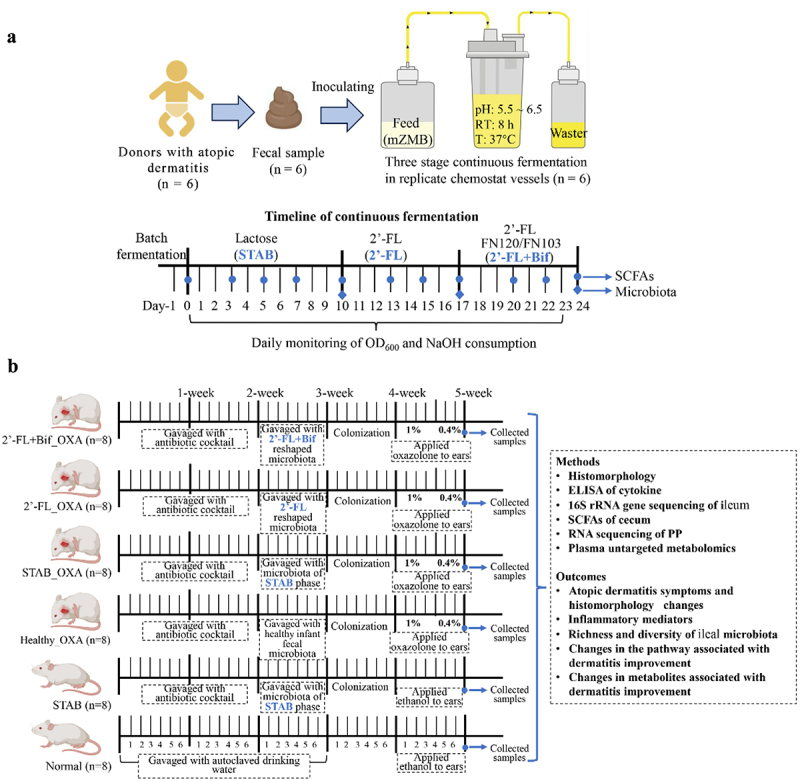
Experimental procedure and timeline. (a) Schematic showing continuous fermentation of ascending colon microbiota. Fecal samples were collected from six infants with atopic dermatitis (AD) and inoculated into six individual bioreactors as fecal slurry. After a 24-h-batch fermentation for system equilibration, continuous fermentation was conducted for 10 d using a medium containing 1% lactose as the carbon source to establish steady-state conditions (STAB). Subsequently, the medium was switched to one containing 1% 2’-fucosyllactose (2’-FL) for additional 7 d of fermentation. A mixture of *Bifidobacterium longum* subsp. *longum* FN103 and *bifidobacterium bifidum* FN120 (1:1, totaling 1 × 10^9^ CFUs) was added daily for the following 7 d. Bacterial density (OD_600_) and NaOH consumption (an indicator of acid production) were monitored daily. Samples of the culture medium were collected at the time points indicated by blue dots and diamonds in the figure to analyze short-chain fatty acids (SCFAs) and bacterial composition. (b) Flow chart of the mouse experiment (*n* = 8 per group). The normal group received no treatment; the STAB group received microbiota from donor 2 at the STAB stage without AD induction; the Healthy_OXA group received microbiota from a healthy infant and AD was induced with OXA; the STAB_OXA, 2’-FL_OXA, and 2’-FL+Bif_OXA groups received microbiota from donor 2 at the end points of the STAB, 2’-FL, and 2’-FL+Bif stages, respectively. All mice were first treated with an antibiotic cocktail for 14 d, then received fecal microbiota transplantation according to the above group for 1 week. After transplantation, mice were normal feed for 1 week to achieve bacterial colonization before AD induction by OXA application. 2’-FL, 2’-Fucosyllactose; AD, atopic dermatitis; bif, mixture of *B. longum subsp. longum longum* FN103 and *B. bifidum* FN120 capable of cross-feeding on 2’-FL; STAB, stabilization stage; OXA, oxazolone.

The *in vitro* continuous fermentation procedure is shown in Figure 1(a). Unfrozen feces were inoculated into lactose-based Zhang – Mills – Block modified medium (mZMB)^19^ (Table S2) in bioreactors of a multifunctional gastrointestinal digestion and fermentation simulator (MGFS, Shangpin Health Technology (Qingdao) Co., Ltd.). Batch fermentation was carried out at 37°C for 24 h with constant oxygen-free nitrogen sparging and stirring to remove oxygen. The setting program for continuous fermentation was as follows: pH 5.5–6.5 (adjusted with 2 mol/L NaOH); the culture medium flow rate was 0.0625 mL/min (retention time was 8 h); the reactor was perfused with an external circulating water bath; and the interlayer temperature was controlled at 37°C. The bacterial density and NaOH consumption were monitored daily, and SCFA levels were determined beginning on the fourth day. The next stage of fermentation was carried out after the indicators stabilized. As shown in Figure 1(a), three experimental stages were set, and the first stage was the stabilization period, the carbon source in mZMB was lactose (STAB, 10 d). The second stage was the 2’-FL intervention period; that is, lactose was changed to 2’-FL (1% for 7 consecutive days, 2’-FL). The third stage was the combined intervention period of bifidobacteria and 2’-FL (2’-FL+Bif); that is, continuous culture with 2’-FL carbon source medium was continued, and a total of 1 × 10^9^ CFUs of FN120 and FN103 (1:1) were added at 10:00 AM (for 7 consecutive days). The fermentation broth was collected 8 h after FN120 and FN103 addition daily and stored at − 80°C for microbiota transplantation in animal experiments.

### 2.4. Microbiota transplantation and OXA-induced AD in mice

The animal experimental protocols were approved by the Animal Ethics Committee of Qingdao University Medical School (QDU-AEC-2023407) and were executed in compliance with the National Guidelines for Experimental Animal Welfare. A total of 48 healthy, 4-week-old male BALB/c mice were procured from Beijing Huafukang Biotechnology Co., Ltd., and maintained in a specific pathogen-free animal facility. Throughout the experiment, the mice were provided sterile mouse chow and sterile drinking water. The environmental conditions were controlled with a relative temperature of 22 ± 1°C, a humidity level of 50 ± 5%, and a 12-hour light‒dark cycle. After 1 week of acclimatization, 48 mice were randomly divided into 6 groups (*n* = 8/group, 4 mice/cage, Figure 1(b)): the Normal group without any treatment (Normal); the negative control group, in which the microbiota of donor 2 at the STAB stage was transplanted but AD was not induced (STAB); the positive control group, in which the microbiota of a healthy infant was transplanted and AD was induced by OXA (Healthy_OXA); the other three experimental groups, in which the microbiota of the other three experimental groups were transplanted with the microbiota from donor 2 at the end points of the STAB stage (STAB_OXA), 2’-FL stage (2’-FL_OXA), or 2’-FL+Bif stage (2’-FL+Bif_OXA), respectively; and then, AD was induced with OXA. The samples from donor 2 were chosen for transplantation because they had the highest levels of SCFAs at the intervention endpoint. Prior to microbiota transplantation, the intestinal microbiota of the mice was depleted by gavage with an antibiotic cocktail: ampicillin 100 mg/kg; metronidazole 100 mg/kg; vancomycin 50 mg/kg; and neomycin sulfate 100 mg/kg for 14 d (100 μL once per day).^20^ Mouse fecal suspensions were spread onto MRS and PTYG agar media for anaerobic and aerobic cultures to confirm the degree of bacterial depletion. Then, mice in specific groups were gavaged with the corresponding bacterial suspension (100 μL, approximately 1 × 10^8^ CFUs per day) according to the design in Figure 1(b) for 7 consecutive days. The Normal and STAB groups were gavaged with PBS. After 7 d of normal feeding to achieve bacterial colonization, the right ears of all the experimental and positive control group mice were smeared with 1% OXA for 4 consecutive days and 0.4% OXA for 3 consecutive days.^21^ The right ears of the Normal and STAB group mice were smeared with ethanol. The thickness of the right ear was measured daily during AD induction, and dermatitis symptoms were recorded for each mouse in the 4 modeling groups. The mice were euthanized on Day 36, and blood was drawn by cardiac puncture into EDTA-coated tubes. Plasma was obtained by centrifuging the samples at 600 × g for 15 minutes at 4°C.

### 2.5. Histopathological analysis

The middle parts of the right ears of 6 mice per group were sampled with a hole punch, fixed in 4% paraformaldehyde, and embedded in paraffin. The tissue was cut into 5 mm sections and stained with hematoxylin and eosin (H&E) or toluidine blue (for mast cells). Mast cells were counted in 10 random fields of view under a light microscope at 200× magnification. The thickness of the dermis was determined in 5 randomly selected fields of view within ear tissue sections.

### 2.6. Determination of cytokines in plasma and ear tissue

Cytokine concentrations, including those of IL-12, immunoglobulin E (IgE), interferon-γ (IFN-γ), and zonulin, were quantified in the plasma of 5 mice per group using enzyme-linked immunosorbent assays (ELISAs; Huijia Biotechnology Co., Ltd., China) adhering to the methodologies outlined for the products. The mouse ear tissues were finely minced and homogenized using a KRH-I model laboratory homogenizer (Shanghai, China) in a phosphate-buffered saline solution (pH 7.3) for 10 seconds. After homogenization, the resulting suspension was centrifuged at 10,000 rpm for 3 minutes. Furthermore, the quantities of thymic stromal lymphopoietin (TSLP), interleukin-4 (IL-4), and IL-33 within homogenized ear tissue were assessed using an analogous ELISA methodology.

### 2.7. RNA sequencing of small intestine Peyer’s patches (PPs)

Collection of PPs and RNA sequencing are described in detail in the Supplementary Methods. The raw fastq files were subjected to quality control (QC) using FastQC. The reads were subsequently aligned to the reference genome GRCm39 release 108, utilizing the corresponding mouse gene annotation file with STAR aligner v2.7.9a. The aligned files were processed to count raw reads using the featureCounts function in the subread package v2.10.5 within the R environment (version 4.2.1). These raw count reads served as the basis for differential gene expression analysis using the limma package in R.

### 2.8. Microbiota analysis

Genomic DNA was isolated from fermentation suspensions and murine ileal contents (5 mice per group) using a QIAamp DNA Stool Mini Kit (Qiagen, Hilden, Germany). DNA quantity and purity were assessed using an Equalbit dsDNA HS Assay Kit. The V3–V4 region of the 16S rRNA gene was amplified by PCR using the forward primer 338F (5′-ACTCCTACGGGAAGCAG-3′) and the reverse primer 806 R (5′-GGACTACHVGGGTWTCTAAT-3′).^22^ The PCR products were purified using an AxyPrep DNA Gel Extraction Kit from Axygen, CA, USA. The purified amplicons were then prepared for sequencing on the Illumina MiSeq platform (Illumina, San Diego, USA) by Biomarker Technologies (China). The raw sequencing data have been submitted to the NCBI Sequence Read Archive under accession numbers PRJNA1186070 for the *in vitro* study and PRJNA1185836 for the *in vivo* study.

### 2.9. SCFA analysis

The determination of SCFAs in the fermentation supernatant and cecal contents of the mice (5 mice per group) was conducted employing gas chromatography spectrometry. The samples (50 mg) were treated with 1 mL of a 50% sulfuric acid solution, vortexed for thorough homogenization, and centrifuged at 13,000 × g for 15 minutes at 4°C. The supernatants were then extracted with 500 μL of ethyl acetate to isolate SCFAs. Analysis of the resulting supernatant was performed on an Agilent 8890 GC/MSD system (Agilent, CA, USA) utilizing an Agilent HP-FFAP column with dimensions of 30 m × 0.25 mm × 0.25 μm. Nitrogen was utilized as the mobile phase gas, flowing at a rate of 1.0 mL per minute with a split ratio of 10:1. The injection port temperature was maintained at 260°C, and the ionization source was maintained at 230°C. The SCFA concentrations were quantified with reference to standard calibration curves.

### 2.10. Nontargeted metabolomic analysis

For 5 mice per group, 100 μL of mouse plasma was vigorously mixed with 750 μL of a 1:1 methanol-ultrapure water mixture, followed by centrifugation at 12,000 rpm for 15 minutes at 4°C. The supernatant was filtered through a 0.45 μm pore size filter to ensure sample clarity. A 10 μL aliquot from each sample was pooled to create a quality control sample. The plasma extracts were analyzed using an Agilent 1290 Infinity II LC system coupled with an Agilent 6530B quadrupole time‒of-flight mass spectrometer. The analysis was performed in positive ion mode via electrospray ionization, following the procedures detailed in the Supplementary methods.

### 2.11. Statistical and bioinformatics analyses

Statistical analyses were conducted using R software, specifically employing the rstatix package. The data are expressed as the means ± SD. For comparisons between two groups, a t test was used for normally distributed data (parametric conditions), whereas the Wilcoxon rank-sum test was used for nonnormally distributed data (nonparametric conditions). Paired data were analyzed using a paired t test or the Wilcoxon signed-rank test, as appropriate. For comparisons among more than two groups, one-way ANOVA with Tukey’s post hoc test was used for parametric data, and a Kruskal‒Wallis test followed by pairwise Wilcoxon rank‒sum tests with Benjamini‒Hochberg correction was applied for nonparametric data. Two-factor repeated-measures ANOVA (Model I) was used to compare the differences between groups for the time series data. For detailed bioinformatics analysis, please see the Supplementary Methods.

## 3. Results

### 3.1. SCFA production by the gut microbiota of infants with AD was enhanced in vitro by a cross-feeding mechanism involving bifidobacteria for the consumption of 2’-FL

**Figure 2.**
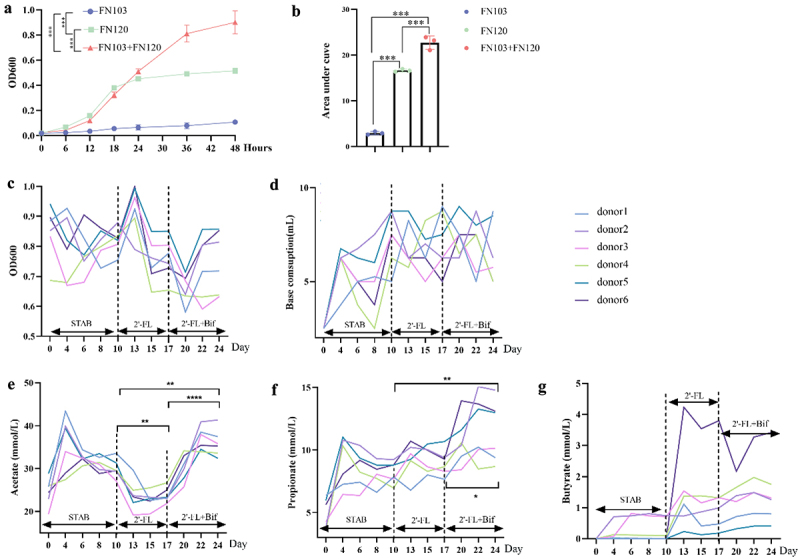
Cosupplementation with 2′-FL and cross-feeding bifidobacteria modulated growth and promoted SCFA production by the microbiota of infants with AD in a continuous fermentation system. (a) Growth curves of *Bifidobacterium longum* subsp. *longum* FN103, *B. bifidum* FN120 and their co-inoculation in MRS medium containing 2’-FL. ***p < 0.001, analyzed by two-way repeated-measures ANOVA (time course); (b) area under the curve of the growth curve, ***p < 0.001, analyzed by one-way ANOVA and Tukey’s post hoc test (*n* = 3/treatment). (c) Bacterial density changes, (d) base consumption (acid production), (e) acetate, (f) propionate and (g) butyrate production during the three stages of fermentation. (c-g) *p < 0.05, **p < 0.01, ***p < 0.001, ****p < 0.0001, analyzed by one-way ANOVA and Tukey’s post hoc test (*n* = 6); 2’-FL, 2’-Fucosyllactose; AD, atopic dermatitis; bif, mixture of *B. longum* subsp. *longum* FN103 and *B. bifidum* FN120 capable of cross-feeding on 2’-FL; STAB, stabilization stage.

To investigate whether *B. bifidum* FN120 and *B. longum* subsp. *longum* FN103 exhibit cross-feeding ability in the metabolism of 2’-FL, we first assessed their growth curves in the presence of 2’−FL. Our data revealed that FN103 lacked the ability to metabolize 2’-FL, whereas FN120 demonstrated independent utilization of 2’-FL (Figure 2(a)), which is consistent with previous reports.^23^ Interestingly, the growth curve of the combined strains was significantly greater than that of FN120 or FN103 alone. The area under the growth curve for the combined strains was increased to 135% of that observed for FN120 alone (*p* < 0.001, Figure 2(b)). The genome annotation based on the carbohydrate-active enzymes database showed that only FN120 contained two GH29 and one GH75 family protein genes involved in 2′-FL catabolism (Table S3). For genes related to HMO transport, FN120 contained 10 and FN103 contained 5 ABC transporter type 1, transmembrane domain MetI-like genes. Each contained one Bacterial extracellular solute-binding protein gene. In addition, only FN103 also contained three ABC transporter-like, ATP-binding domain genes (Table S4). The protocol outlined in Figure 1(a) was subsequently employed to investigate the impact of 2’-FL and bifidobacterial cosupplementation on the *in vitro* ascending colonic microbiota of 6 infants with AD. The optical densities of the cultures (Figure 2(c)) and the acetate (Figure 2(e)), propionate (Figure 2(f)), and butyrate (Figure 2(g)) levels stabilized at the 8^th^ day of continuous fermentation, although NaOH consumption still fluctuated (Figure 2(d)).

Acetate was the most abundant SCFA produced by the microbiota, followed by propionate and butyrate. At the end of the STAB stage, the acetate concentration peaked at 30.06 ± 1.98 mmol/L. During the 2’-FL treatment, acetate production swiftly declined to a stable state after 3 d to 79.33% of the STAB endpoint (*p* < 0.05). Following *Bifidobacterium* addition, acetate levels progressively increased, plateauing after 5 d at 119.67% of the STAB endpoint (*p* < 0.0001), and were higher than those at the 2’-FL intervention endpoint (*p* < 0.01). The propionate level after intervention with 2’-FL+Bif was 140.02% higher than that at the STAB endpoint (*p* < 0.01) and 128% greater than that after 2’-FL intervention (*p* < 0.05). Donor 2 had the highest acetate and propionate production when supplemented with 2’-FL and bifidobacteria. The butyrate levels increased in a donor-dependent manner following 2’−FL and 2’-FL+Bif supplementation. Therefore, the microbiota of infants with AD exhibited a limited capacity to utilize 2’-FL to produce SCFAs, as previously reported.^24,25^ It would have been interesting to report the composition of the microbiota of these infants. A significant increase in acetate and propionate production was observed after 2’-FL+Bif treatment.

### 3.2. The gut microbiota of infants with AD was reshaped by 2’-FL and cross-feeding bifidobacteria in vitro

**Figure 3.**
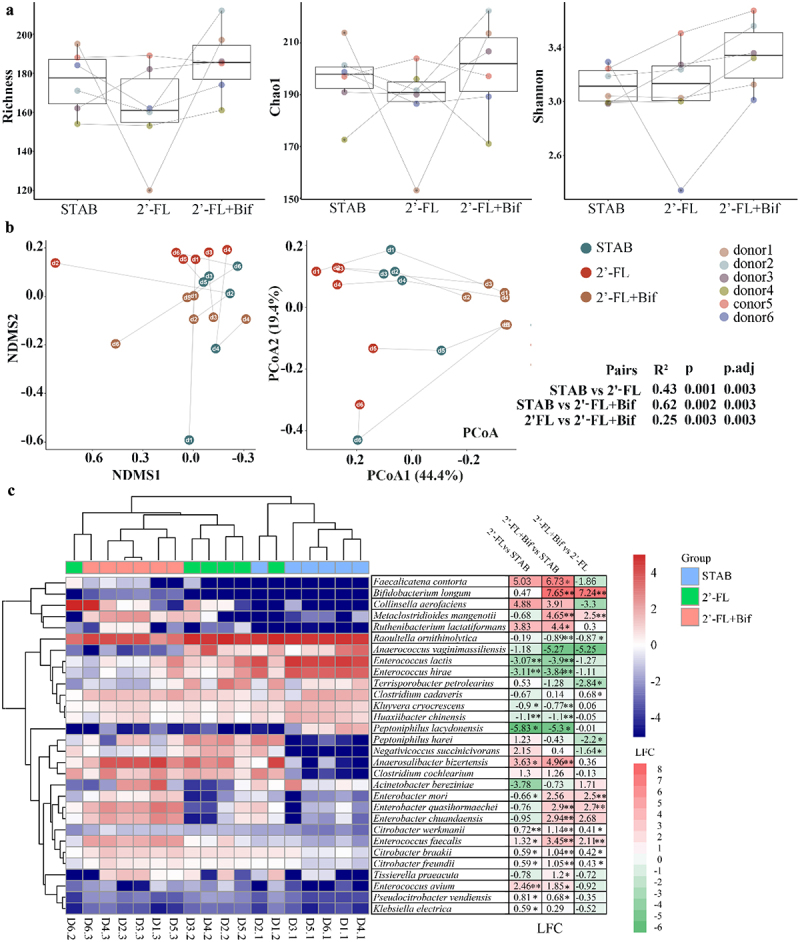
Cosupplementation with 2′-FL and cross-feeding bifidobacteria reshaped the microbiota of infants with AD *in vitro*. (a) α diversity; (b) β diversity; (c) heatmap of species on the log scale. The data are the relative abundance (x) of the species transformed by log2(x + 0.0005). The mean log2-fold change (LFC) is listed to the right of the species names, *p < 0.05, **p < 0.001 analysed by Welch’s t test. 2’-FL, 2’-fucosyllactose; AD, atopic dermatitis; bif, mixture of *Bifidobacterium longum subsp. longum* FN103 and *bifidobacterium bifidum* FN120 capable of cross-feeding on 2’-FL; NDMS, nonmetric multidimensional scaling; PCoA, principal coordinate analysis; STAB, stabilization stage.

We then investigated whether 2’-FL and cross-feeding bifidobacterial cosupplementation reshaped the infant microbiota structure. As depicted in Figures 3(a), 2’-FL supplementation induced a significant donor-dependent decrease in microbial richness. Upon the introduction of two *Bifidobacterium* strains, increases in richness were noted for all donors except donor 5. The Chao1 richness estimator was lower than the STAB endpoint post-2’-FL supplementation. Furthermore, bifidobacterial supplementation led to increasing trends in the Chao1 index for all donors except donor 5. Cosupplementation with 2’-FL and cross-feeding bifidobacteria increased the Shannon indices across all donors. However, these changes in the three α diversity indices did not reach statistical significance between the groups.

Statistically significant differences were detected among all three groups (*p.adj* = 0.003, Figure 3(b)) based on the Bray‒Curtis distance (Table S5 and S6). NMDS ordination based on Bray‒Curtis distances (Figure 3(b)) revealed a significant shift in the microbial community structure away from the STAB phase after 2’−FL supplementation. 2’-FL and bifidobacterial supplementation induced a further shift in the microbial community structure to the opposite extent from that of STAB. 2’−FL and bifidobacteria supplementation subsequently reduced the dispersion among samples and shifted them toward the opposite side of the 2’-FL-supplemented group, centered on STAB, with the X-axis representing 44.4% of the variance explained.

According to Figure 3(c), the species profile of samples treated with 2’-FL+Bif formed a distinct cluster, characterized by significant increases in the relative abundances of *Faecalicatena contorta* (LFC = 6.73, *p* < 0.05), *B. longum* (LFC = 7.65, *p* < 0.001), *Metaclostridioides mangenotii* (LFC = 4.65, *p* < 0.001), and *Ruthenibacterium lactatiformans* (LFC = 4.4, *p* < 0.05).

Our findings indicated that the dysbiotic gut microbiota in infants with AD can be reshaped by 2’-FL and cross-feeding with bifidobacterial cosupplementation, as evidenced by an increased relative abundance of bacterial strains with high SCFA-producing potential.

### 3.3. AD was prevented in OXA-induced mice transplanted with microbiota reshaped by 2’-FL and cross-feeding bifidobacteria

**Figure 4.**
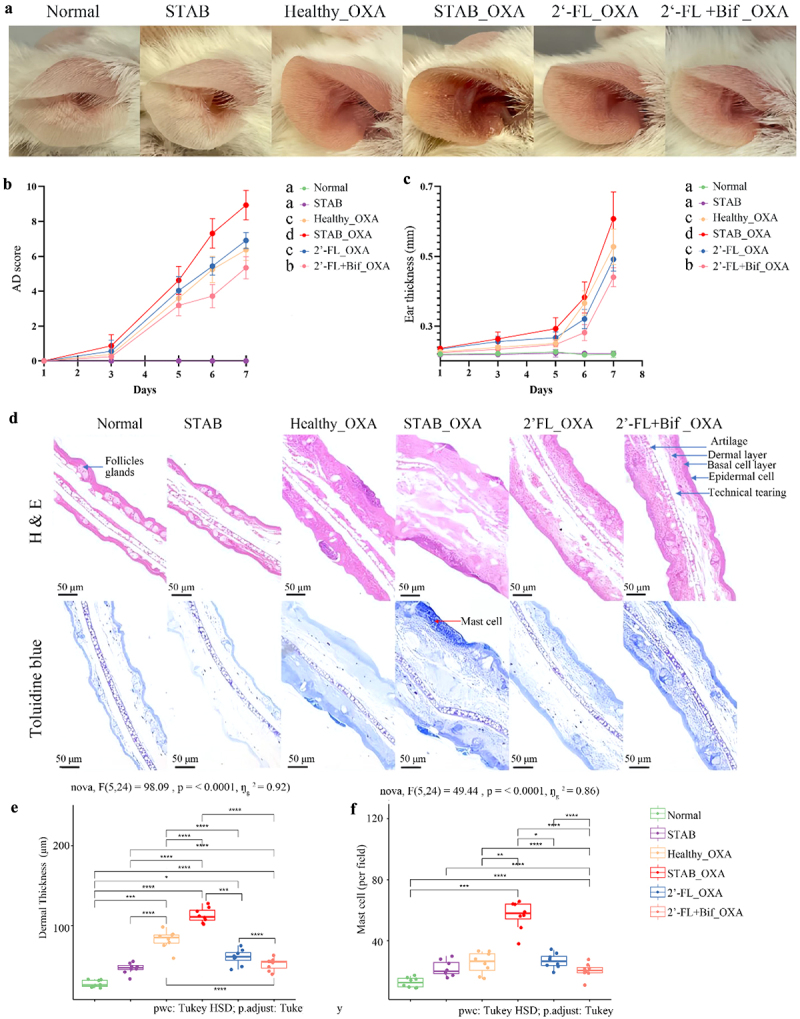
Severity of AD induced by OXA in mice transplanted with microbiota from continuous fermentation *in vitro*. (a) Clinical features of mouse ears on day 35; (b) Atopic dermatitis symptom scoring (*n* = 8); (c) changes in mouse ear thickness (*n* = 8). (d) Typical tissue sections stained with hematoxylin and eosin (H&E) and toluidine blue. (e) Thickness of the dermis in any 5 randomly selected fields of view within ear tissue sections. (f) Number of mast cells in any 10 randomly selected fields of view within ear tissue sections. The data are presented as the means ± SD (*n* = 8/group); different lowercase letters indicate differences (*p* < 0.001) according to two-way repeated-measures ANOVA (time course). Asterisks indicate significance levels of *p < 0.05; **p < 0.01; ***p < 0.001; ****p < 0.0001 according to one-way ANOVA and Tukey’s multiple comparisons. 2’-FL_OXA, 2’-FL+Bif_OXA, STAB_OXA, Healthy_OXA, transplantation of *in vitro* microbiota supplemented with 2’-FL, 2’-FL+Bif, that at the STAB stage, and of fecal microbiota from healthy infants, respectively, and induction of AD with OXA; AD, atopic dermatitis; bif, *Bifidobacterium longum* subsp. *longum* FN103 and *bifidobacterium bifidum* FN120 capable of cross-feeding on 2’-FL; normal, healthy mouse control; OXA, oxazolone; STAB, mice transplanted with stabilization stage fecal microbiota of donor 2 without AD induction.

We next validated the modulatory effect of the gut microbiota reshaped by 2’-FL and cross-feeding bifidobacteria on OXA-induced AD in mice (Figure 1(b)). The mice were treated with a cocktail of broad-spectrum antibiotics for 14 d to deplete the commensal microbiota before microbiota transplantation. To better understand the effects of cosupplementation with 2’-FL and bifidobacteria, we performed microbiota transplantation from the STAB phase, 2’−FL or 2’-FL+Bif treatment endpoints. Fecal culture samples from donor 2 were chosen for transplantation because they had the highest levels of SCFAs at the intervention endpoint, as described previously (Figure 2(e-f)). AD was subsequently induced using OXA following the protocol outlined in Figure 1(b). The results indicated that no dermatitis symptoms were observed in the ears of the mice in the Normal group or in those receiving STAB-phase microbiota transplantation without OXA application (Figure 4(a,b)), and there was no trend toward ear thickening during the experimental period (Figure 4(c)). Notably, at the end of the experiment, no abnormalities or mast cell infiltration were observed in the ear sections of those mice (Figure 4(d)), and no thickening of the dermis or increase in mast cells was observed under microscopic examination (Figure 4(e-f)). These findings suggested that, in the absence of OXA induction, gut microbiota dysbiosis alone did not directly cause AD.

As depicted in Figure 4(a), among the four groups of mice with AD induced by OXA, the STAB_OXA group displayed typical symptoms of AD, including red and swollen ears with desquamation. The mice in the 2’-FL+Bif_OXA group only exhibited mild ear thickening, and no significant redness or swelling was observed (Figure 4(a)), with symptoms and ear thickness significantly greater than those in the other three groups (Figure 4(b,c), *p* < 0.01). Compared with those in the STAB_OXA group, the Healthy_OXA and 2’-FL_OXA groups of mice presented mild redness and swelling (Figure 4(a)), with symptoms significantly improved (*p* < 0.01).

H&E staining (Figure 4(d)) revealed that the mice in the STAB_OXA group presented the most severe dermal swelling, sebaceous gland atrophy, and an incomplete epidermal layer. Compared with that in the STAB_OXA group, the dermal thicknesses in the Healthy_OXA and 2’-FL_OXA groups were lower; however, sebaceous gland atrophy persisted, and the epidermal layer in the Healthy_OXA group remained incomplete. In the 2’-FL+Bif_OXA group, dermal swelling was markedly diminished, the epidermis was intact, and sebaceous glands showed appropriate atrophy. The average dermal thicknesses of the four groups of mice decreased in the order of STAB_OXA, Healthy_OXA, 2’-FL_OXA, and 2’-FL+Bif_OXA, with significant differences between the groups (*p* < 0.001, Figure 4(d)). The average dermal thicknesses in the 2’-FL_OXA and 2’-FL+Bif_OXA groups were only 53.54% and 45.80% that in the STAB_OXA group, respectively (Figure 4(e)). As shown in Figure 4(f), the average number of mast cells in the ear tissue of the STAB_OXA group was 223.15% greater than that in the Healthy_OXA group (*p* < 0.001). The average mast cell counts in the 2’-FL_OXA and 2’-FL+Bif_OXA groups were only 47.24% and 36.20% of those in the STAB_OXA group, respectively (*p* < 0.001).

The number of PPs in the small intestine of the mice was also compared, as this metric is closely related to the immune development of the mice. Figure S2 shows that the number of PPs in the 2’-FL+Bif_OXA group (7 ± 2, *p* < 0.001) was significantly greater than that in the STAB_OXA group (3 ± 1).

Overall, these data revealed that cosupplementation with 2’-FL and cross-feeding bifidobacteria was more effective at reducing the susceptibility of the gut microbiota to dermatitis in infants with AD than supplementation with 2’−FL alone.

### 3.4. Ad-related factors were improved by transplanting the microbiota reshaped by 2’-FL and cross-feeding bifidobacteria in oxa-induced mice

**Figure 5.**
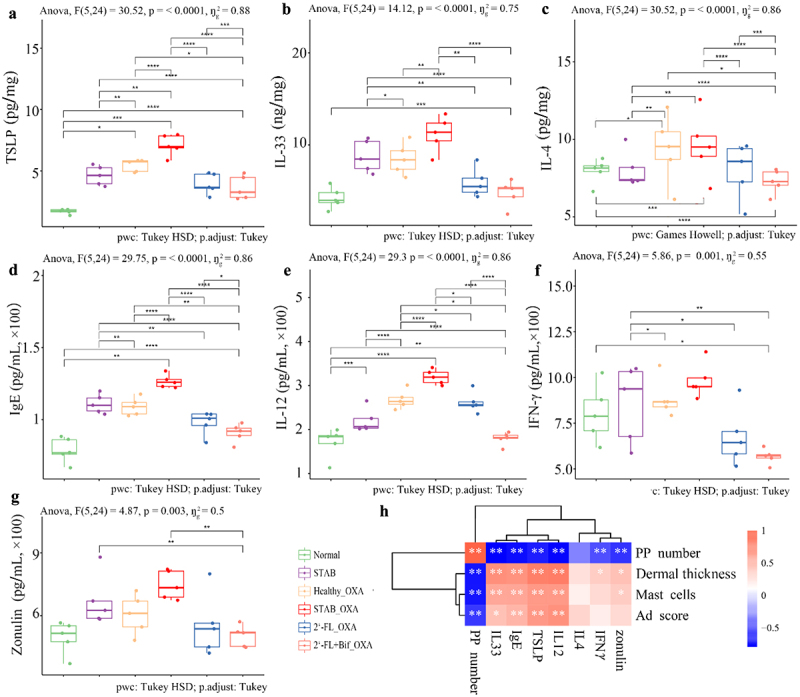
Ad-related biomarkers in ear homogenates and plasma of oxa-sensitized mice transplanted with microbiota from continuous fermentation *in vitro*. (a) Ear thymic stromal lymphopoietin (TSLP); (b) ear IL-33; (c) ear IL-4; (d) plasma total immunoglobulin E (IgE); (e) plasma IL-12; (f) plasma interferon gamma (ifn-γ); (g) plasma zonulin. (h) Correlations between AD indicators and immune indicators. The data are presented as the means ± SD (*n* = 5 mice/group) for (A-G), and asterisks indicate significance levels of *p < 0.05; **p < 0.01; ***p < 0.001; ****p < 0.0001 according to one-way ANOVA and Tukey’s multiple comparisons. The bar for the heatmap indicates the color legend of the Spearman correlation coefficients; *p < 0.05, **p < 0.01 indicate *p* values calculated for each pair of samples in the matrix. 2’-FL_OXA, 2’-FL+Bif_OXA, STAB_OXA, Healthy_OXA, transplantation of *in vitro* microbiota supplemented with 2’-FL, 2’-FL+Bif, that at the STAB stage, and of fecal microbiota from healthy infants, respectively, and induction of AD with OXA; AD, atopic dermatitis; bif, *Bifidobacterium longum* subsp. *longum* FN103 and *bifidobacterium bifidum* FN120 capable of cross-feeding on 2’-FL; normal, healthy mouse control; OXA, oxazolone; STAB, mice transplanted with stabilization stage fecal microbiota of donor 2 without AD induction.

Elevated levels of total plasma IgE and Th2 and Th1 cytokines play important roles in inflammation and hypertrophy of the skin in AD.^26^ The relevant cytokines in the plasma and ear tissue homogenate were detected to explore the associations between microbiota transplantation and the improvement of dermatitis. Compared with those in the Normal group, the levels of TSLP and IL-4 in the ears of the mice in the STAB_OXA group were significantly greater (*p* < 0.01), and the level of IL-33 also markedly increased (Figure 5(a-c)). Compared with those in the STAB_OXA group, the ears of the Healthy_OXA group presented significantly lower levels of TSLP and IL-33 (*p* < 0.05), indicating that gut microbiota dysbiosis increases susceptibility to inflammation induced by OXA. The levels of these three cytokines in the 2’-FL_OXA and 2’-FL+Bif_OXA groups were significantly lower than those in the STAB_OXA group (*p* < 0.01). Notably, the levels of TSLP and IL-4 in the 2’-FL+Bif_OXA group were significantly lower than those in the Healthy_OXA group (*p* < 0.05). The total IgE and IL-12 levels in the plasma of the STAB_OXA group mice were significantly greater than those in the other three groups of OXA-induced mice (*p* < 0.01). Notably, the levels of these two factors in the 2’-FL+Bif_OXA group were significantly lower than those in the 2’-FL_OXA group and the Healthy_OXA group (*p* < 0.05). Furthermore, the plasma zonulin and IFN-γ levels in all OXA-induced mice were not significantly greater than those in Normal mice, although there was a trend toward increased levels in the STAB_OXA group. These findings suggested that intestinal mucosal barrier damage and Th1 immune polarization were not the main contributors to increased inflammatory susceptibility. IL-33 and TSLP levels in ear homogenates, along with IgE and IL-12 levels in plasma, were significantly positively correlated with AD scores, ear dermal thickness, mast cell counts in ear sections, and PP counts (*p* < 0.01, Figure 5(h)).

In summary, microbiota transplantation experiments revealed that the gut microbiota of infants with AD has a greater capacity to induce allergy-related cytokines than that of healthy infants. Transplantation of the microbiota remodeled by 2’-FL and cross-feeding bifidobacteria reduced the Th2 cytokines in mice to levels even lower than those in mice transplanted with microbiota from healthy infants.

### 3.5. SCFA promotion and alterations in the ileal microbiota are related to AD prevention

**Figure 6.**
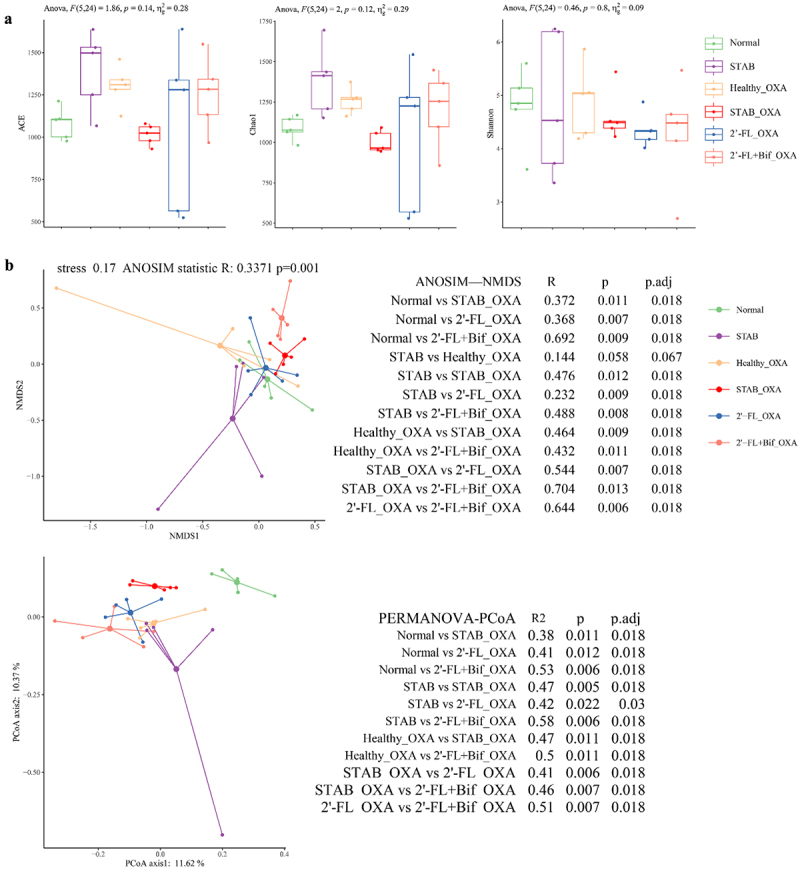
α and β diversities of the ileal microbiota of oxa-sensitized mice transplanted with microbiota from continuous fermentation *in vitro*. (a) α diversity indicated by the ACE, Chao1 and Shannon indices; (b) β diversity determined by nonmetric multidimensional scaling with ANOSIM (analysis of similarities, NDMS-ANOSIM) and principal coordinate analysis of the bray‒curtis distance with pairwise permutational multivariate analysis of variance (permanova‒PCoA). The microbiota was analyzed by 16S rRNA gene amplicon sequencing (v3-v4). 2’-FL_OXA, 2’-FL+Bif_OXA, STAB_OXA, Healthy_OXA, transplantation of *in vitro* microbiota supplemented with 2’-FL, 2’-FL+Bif, that at the STAB stage, and of fecal microbiota from healthy infants, respectively, and induction of AD with OXA; AD, atopic dermatitis; bif, *Bifidobacterium longum* subsp. *longum* FN103 and *bifidobacterium bifidum* FN120 capable of cross-feeding on 2’-FL; normal, healthy mouse control; OXA, oxazolone; STAB, mice transplanted with stabilization stage fecal microbiota of donor 2 without AD induction.

Ileal bacteria regulate host immunity to contribute to allergic sensitization.^27^ The prevention of AD in the aforementioned microbiota-transplanted mice may be associated with the specific structure and metabolites of the gut microbiota; hence, the ileal microbiota was subjected to amplicon sequencing analysis. SCFAs were also analyzed. As illustrated in Figure 6(a), both the ACE and Chao1 indices of the microbiota in the 2’-FL_OXA and 2’-FL+Bif_OXA groups tended to be greater than those in the STAB_OXA group. Bray‒Curtis distance was the best choice for the PCoA of the microbiota in the present study (R^2^ value of 0.29, Table S7), with dispersion not significantly different among the three groups (Table S8). The stress value of the NMDS analysis was 0.17 (Figure 6(b)), indicating an acceptable fit of the NMDS model. ANOSIM of NMDS revealed significant differences among the 2’-FL+Bif_OXA group and the STAB_OXA (*R* = 0.704), Normal (*R* = 0.692), and 2’-FL_OXA groups (*p* < 0.05). Additionally, significant differences were observed between the 2’-FL_OXA group and the STAB_OXA, Normal, and STAB groups (*R* < 0.5, *p* < 0.05). PCoA based on Bray – Curtis distance indicated that PCoA1 accounted for 11.62% of the intergroup differences, with the Normal group significantly diverging from the other groups (*p.adj* < 0.05). Within the other groups, STAB_OXA notably deviated from the remaining samples, particularly from the STAB group. One sample in the STAB group diverged from the others (*p.adj* < 0.05), contributing the most to the variation in PCoA2 (10.37%).

**Figure 7.**
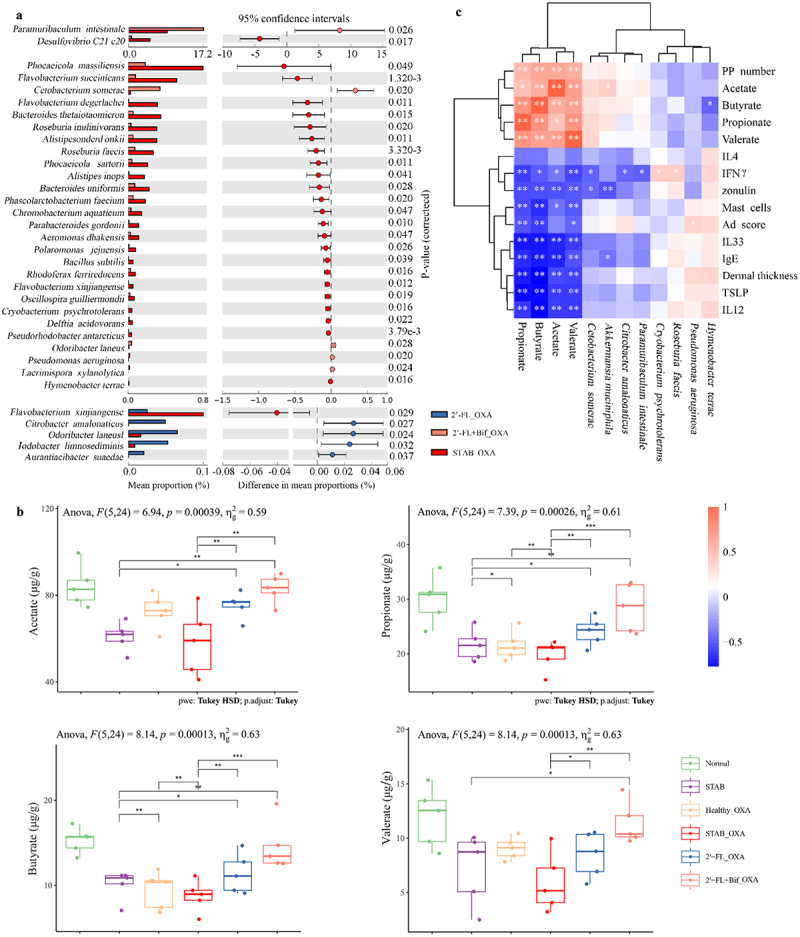
Different bacterial species in the ileum and cecal SCFA levels of oxa-sensitized mice transplanted with microbiota from continuous fermentation *in vitro*. (a) Differential bacterial species in the ileum identified by STAMP analysis. Statistical significance (*p* < 0.05) was detected by Welch’s t test. (b) Cecal SCFA levels. (c) Correlations between AD indicators and ileum species and cecal SCFAs. Asterisks in (b) indicate a significance level of *p < 0.05, **p < 0.01, and ***p < 0.001 according to one-way analysis of variance, followed by individual comparisons with Tukey–Kramer’s post hoc test. The bar for the heatmap indicates the color legend of the Spearman correlation coefficients; *p < 0.05, **p < 0.01 indicate *p* values calculated for each pair of samples in the matrix. 2’-FL_OXA, 2’-FL+Bif_OXA, STAB_OXA, Healthy_OXA, transplantation of *in vitro* microbiota supplemented with 2’-FL, 2’-FL+Bif, that at the STAB stage, and of fecal microbiota from healthy infants, respectively, and induction of AD with OXA; AD, atopic dermatitis; bif, *Bifidobacterium longum* subsp. *longum* FN103 and *bifidobacterium bifidum* FN120 capable of cross-feeding on 2’-FL; normal, healthy mouse control; OXA, oxazolone; SCFA, short-chain fatty acid; STAB, mice transplanted with stabilization stage fecal microbiota of donor 2 without AD induction.

As shown in Figure 7(a), the relative abundances of *Paramuribaculum intestinale*, *Cetobacterium somerae*, *Odorbacter laneus*, and *Pseudomonas aeruginosa* in the ileum of 2’-FL+Bif_OXA group mice were significantly greater than those in the STAB_OXA group (*p* < 0.05), with 24 species showing significantly lower relative abundances than those in the STAB_OXA group (*p* < 0.05). In contrast, when the 2’-FL_OXA group was compared with the STAB_OXA group, only four species exhibited significant changes in relative abundance (*p* < 0.05). In the 2’-FL+Bif_OXA group, a total of 1791 ASVs were identified in the ileum, 374 of which were shared with the microbiota of the donor (Figure S3). Among these ASVs, *Akkermansia muciniphila*, *Heminiphilus faecis*, and *Ligilactobacillus murinus* were the predominant species common to this group of mice (Figure S2) and were the dominant species that successfully colonized the ileum of the mice from the transplanted microbiota treated with 2’−FL and bifidobacteria.

The levels of cecal acetate, propionate, and butyrate in the 2’-FL_OXA and 2’-FL+Bif_OXA groups were significantly elevated compared with those in the STAB and STAB_OXA groups (*p* < 0.05, Figure 7(b)), with only the 2’-FL+Bif_OXA group levels nearing those of the Normal group. The concentrations of cecal propionate and butyrate in the STAB_OXA group were markedly lower than those in the Healthy_OXA group (*p* < 0.01). The levels of cecal valerate in the 2’-FL_OXA and 2’-FL+Bif_OXA groups were both significantly greater than those in the STAB_OXA group (*p* < 0.05), and only the 2’-FL+Bif_OXA group levels were significantly greater than those in the STAB group (*p* < 0.01), achieving the level observed in the Normal group.

Spearman’s correlation analysis was employed to explore the potential contributions of the aforementioned differential bacterial species, dominant transplanted and colonizing species, and SCFAs to AD. As shown in Figure 7(c), propionate, butyrate, and valerate were significantly negatively correlated with multiple other AD markers except for ear IL-4 (*r* > 0.5, *p* < 0.05). In addition to the IL-4 and AD scores, acetate was also significantly negatively correlated with other AD markers (*r* > 0.45, *p* < 0.05). Additionally, the relative abundance of ileal *A. muciniphila* was negatively correlated with plasma IgE (*r* = −0.37, *p* < 0.05) and zonulin (*r* = −0.48, *p* < 0.01) and positively correlated with acetate (*r* = 0.39, *p* < 0.05). The relative abundances of *Roseburia faecis* (*r* = 0.40, *p* < 0.05) and *Cryobacterium psychrotolerans* (*r* = 0.38, *p* < 0.05) were positively correlated with the plasma IFN-γ level. The relative abundances of *Citrobacter amalonaticus* (*r* = −0.44, *p* < 0.05), *Para. intestinale* (*r* = −0.44, *p* < 0.05), and *C. somerae* (*r* = −0.43, *p* < 0.05) were negatively correlated with IFN-γ concentration. Notably, the relative abundance of *P. aeruginosa* was the only factor significantly correlated with the AD score (*r* = 0.36, *p* < 0.05).

In summary, the microbiota reshaped by 2’-FL and cross-feeding bifidobacteria exhibited a stronger SCFA-promoting effect than that reshaped by 2’-FL alone, characterized by an increased abundance of the ileum SCFA-producing bacterium *Para. intestinale* ^28^ and a decreased relative abundance of the potential AD-inducing bacterium *P. aeruginosa*.

### 3.6. Retinol metabolism and the relative abundance of tolerant immune cells were increased in the PPs of OXA-induced mice transplanted with microbiota reshaped by 2’-FL and cross-feeding bifidobacteria.

**Figure 8.**
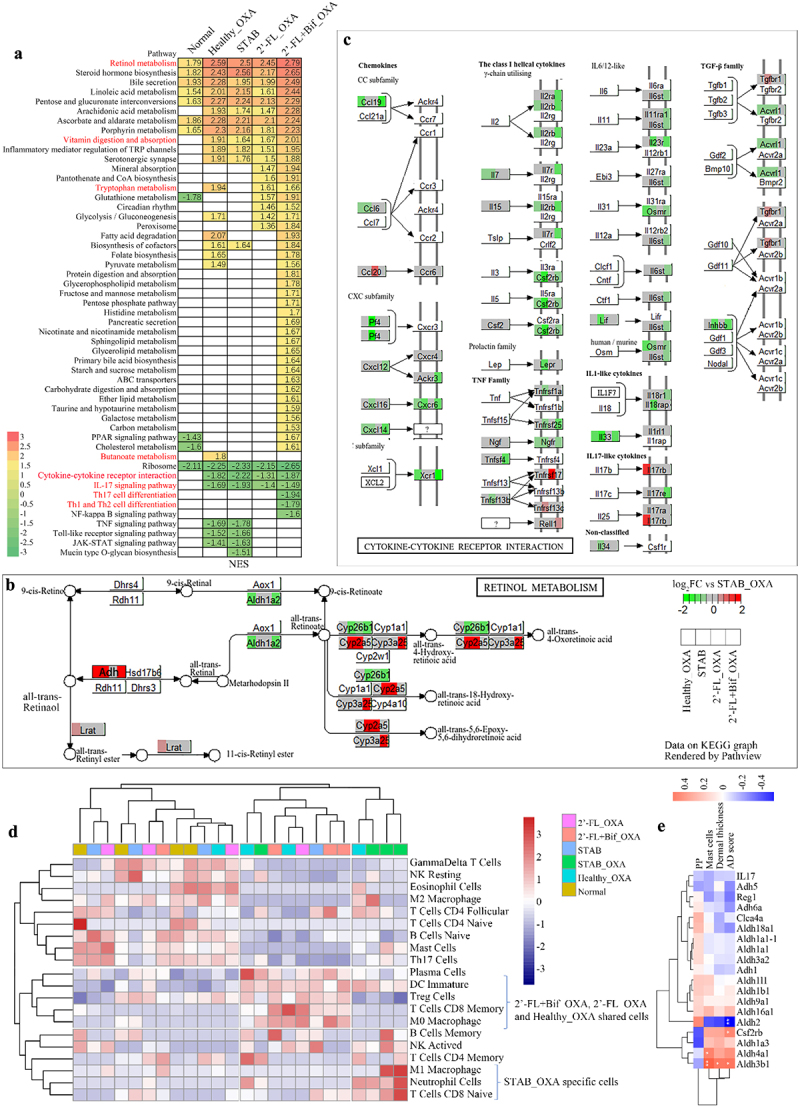
Gene expression of PPs in oxa-sensitized mice determined by bulk RNA sequencing. (a) KEGG enrichment results of DEGs relative to the STAB_OXA group with NES values greater than 1.5. (b) Expression of genes in the retinol metabolism pathway mapped using Pathview relative to the STAB_OXA group. (c) Expression of genes in the cytokine‒cytokine receptor interaction pathway mapped using Pathview relative to the STAB_OXA group. (d) Hierarchical clustering of immune infiltration analysis of 22 immune cell subtypes in PPs using the ward clustering algorithm and Euclidean distance measurements. Gene expression is expressed as the log2FC relative to the normal group. 2’-FL_OXA, 2’-FL+Bif_OXA, STAB_OXA, Healthy_OXA, transplantation of *in vitro* microbiota supplemented with 2’-FL, 2’-FL+Bif, that at the STAB stage, and of fecal microbiota from healthy infants, respectively, and induction of AD with OXA; AD, atopic dermatitis; bif, *Bifidobacterium longum* subsp. *longum* FN103 and *bifidobacterium bifidum* FN120 capable of cross-feeding on 2’-FL; normal, healthy mouse control; OXA, oxazolone; FC, fold change; DEG: differentially expressed gene; KEGG: Kyoto Encyclopedia of genes and Genomes; NES, normalized enrichment scores; STAB, mice transplanted with stabilization stage fecal microbiota of donor 2 without AD induction.

Oral tolerance plays a protective role in the development of AD,^29^ and PPs have been shown to be essential for oral tolerance.^30^ RNA sequencing of PPs was employed to further analyze whether the SCFA promotion and ileum microbiota modification observed above could induce an immune tolerance response. Figure 8(a) shows that, compared with those in the STAB_OXA group, the most upregulated pathway in the 2’-FL+Bif_OXA group among the differentially expressed enriched KEGG pathways was retinol metabolism according to the normalized enrichment score (NES = 2.79), whereas the most downregulated pathways were the IL-17 signaling pathway (NES = −1.47), Th17 cell differentiation (NES = −1.94), Th1 and Th2 cell differentiation (NES = −1.79), and cytokine‒cytokine receptor interaction (NES = −1.87). Pathview mapping of the retinol metabolism pathway (Figure 8(b)) revealed significant upregulation of alcohol dehydogenase gene (ADH4) expression in the Healthy_OXA (log_2_FC = 1.92), STAB (log_2_FC = 1.60), and 2’-FL+Bif_OXA (log_2_FC = 1.62) groups, which converted retinol into retinoic acid (RA), relative to the STAB_OXA (*p* < 0.001) group. The expression of aldehyde dehydrogenase 1 family member L1 (Adh1), another enzyme that converts retinol to RA, was also specifically upregulated in the 2’-FL+Bif_OXA group (log_2_FC = 1.63, *p* < 0.001). Figure 8(e) shows that the expression of Aldh2 was significantly negatively correlated with the AD score (*p* < 0.01). The cytokine‒cytokine receptor interaction pathway diagram (Figure 8(c)) revealed that the expression levels of several genes related to cytokine receptors in the CXE subfamily, class 1 helical cytokines, IL6/12-like, and IL-17 receptors were specifically downregulated in the 2’-FL+Bif_OXA group. Deconvolution of the RNA sequencing data revealed the immune cell composition of the PPs. As depicted in Figure 8(d), the PPs of the mice in the STAB_OXA group were enriched with M1 macrophages, neutrophils, and naive CD8 T cells. In contrast, the cells enriched in the 2’-FL_OXA, 2’-FL+Bif_OXA, and Healthy_OXA groups were immature dendritic cells (DCs), regulatory T cells (Tregs), CD8 memory T cells, and M0 macrophages.

Overall, the mice in the 2’-FL-Bif_OXA group indeed exhibited an immunotolerant response characterized by the induction of retinol metabolism; the suppression of inflammatory cytokines and receptor pathways; and the increased abundance of tolerance-related immune cells.

### 3.7. Plasma retinoate was specifically upregulated in oxa-induced mice transplanted with 2’-FL, and cross-feeding bifidobacteria remodeled the microbiota

**Figure 9.**
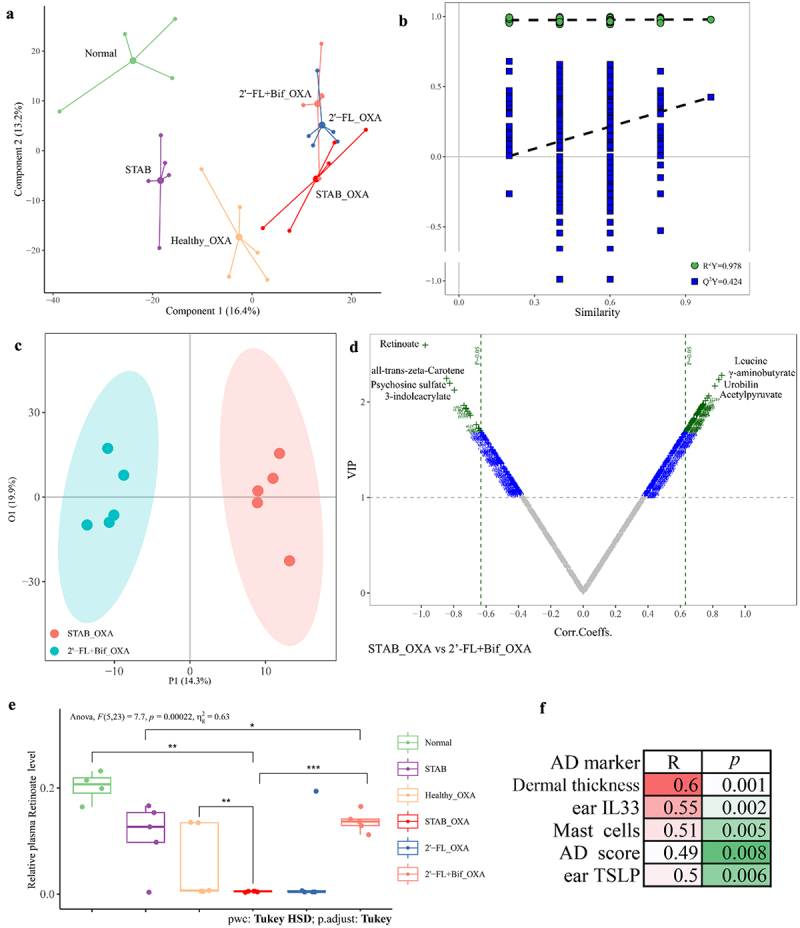
Plasma metabolites of oxa-sensitized mice analyzed following transplantation with microbiota from continuous *in vitro* fermentation. (A) Score plot based on partial least squares discriminant analysis (PLS-DA). (B, C and D) permutation plot, score plot and V-plot of established orthogonal PLS-DA (OPLS-DA) between the 2’-FL+Bif_OXA and STAB_OXA groups, respectively. Permutation analysis plotting R^2^ and Q^2^ from 200 permutation tests in the OPLS-DA model. The y-axis shows R^2^ and Q,^2^ whereas the x-axis shows the correlation coefficient of permuted and observed data. The two points on the right represent the observed R^2^ and Q^2^ values. The cluster of points on the left represents 200 permuted R^2^s and Q^2^s. The green and blue dots represent the R^2^ and Q^2^ values, respectively. The dashed lines denote the corresponding fitted regression lines for the observed and permutated R^2^ and Q^2^ values. (E, F) relative level of plasma retinoate and its correlation with ad-related markers. Asterisks in (B) indicate significance levels of *p < 0.05, **p < 0.01, and ***p < 0.001 according to one-way analysis of variance, followed by individual comparisons with Tukey–Kramer’s post hoc test. 2’-FL_OXA, 2’-FL+Bif_OXA, STAB_OXA, Healthy_OXA, transplantation of *in vitro* microbiota supplemented with 2’-FL, 2’-FL+Bif, that at the STAB stage, and of fecal microbiota from healthy infants, respectively, and induction of AD with OXA; AD, atopic dermatitis; bif, *Bifidobacterium longum* subsp. *longum* FN103 and *bifidobacterium bifidum* FN120 capable of cross-feeding on 2’-FL; normal, healthy mouse control; OXA, oxazolone; STAB, mice transplanted with stabilization stage fecal microbiota of donor 2 without AD induction. VIP, variable importance in projection.

To demonstrate that changes in the gut microbiota and mucosal immune responses can influence the immune status of distant tissues through the circulation, we further conducted a nontargeted metabolomic analysis of plasma. According to the PLS-DA plot (Figure 9(a)), 2’-FL+Bif_OXA and 2’-FL_OXA tended to separate from STAB_OXA, but STAB and Healthy_OXA tended to separate from each other. OPLS-DA was employed to analyze the differences in plasma metabolites between the 2’-FL+Bif_OXA and STAB_OXA groups. A Q^2^ value of 0.424 was acceptable, and an R^2^ value of 0.978 indicated good fit accuracy (Figure 9(b)). The OPLS-DA score plot revealed clear and separate clustering between the 2’-FL+Bif_OXA and STAB_OXA groups, which was explained by the X-axis (14.3%) (Figure 9(c)).

According to the variable importance in projection (VIP) Vplot (Figure 9(d)), the plasma metabolites presented at the highest concentrations in the 2’-FL+Bif_OXA group were retinoate (VIP = 2.06, *p* < 0.001), (VIP = 2.23, *p* < 0.01), psychosine sulfate (VIP = 2.18, *p* < 0.01), and 3-indoleacrylate (VIP = 2.11, *p* < 0.01). In contrast, the STAB_OXA group presented the highest relative concentrations of leucine (VIP = 2.26, *p* < 0.01), γ-aminobutyrate (VIP = 2.22, *p* < 0.01), urobilin (VIP = 2.15, *p* < 0.01), and acetylpyruvate (VIP = 2.05, *p* < 0.01). Among them, retinoate had a significantly lower relative content in the STAB_OXA group than in the Normal and Healthy_OXA groups (*p* < 0.01, Figure 9(e)). The retinoate level in the 2’-FL+Bif_OXA group was significantly greater than that in the STAB_OXA group (*p* < 0.001), approaching the level of the Normal group. Moreover, the relative level of retinoate was significantly positively correlated with ear dermal thickness, IL33, mast cells, TSLP, and the terminal AD score (*R* ≥ 0.5, *p* < 0.01; Figure 9(f)). Therefore, this study demonstrated that after the transplantation of microbiota reshaped by 2’-FL and bifidobacteria, the retinoic acid production response induced in small intestine PPs was also observed in the circulatory system, indicating that the immune tolerance of distal tissues can also be shaped.

## 4. Discussion

We demonstrated that *B. bifidum* FN120 and *B. longum* subsp. *longum* FN103, derived from human milk, efficiently utilized 2’-FL through cross-feeding. This cross-feeding mechanism allowed these two *Bifidobacterium* strains, when supplemented alongside 2’-FL, to effectively reshape the structure of the gut microbiota in infants with AD *in vitro*, leading to an increase in the production of SCFAs. Transplanting this reshaped microbiota *in vitro* into mice previously treated with a cocktail of broad-spectrum antibiotics to deplete their own endogenous microbiota effectively prevented OXA-induced AD. One potential mechanism involved reshaping the microbiota in the small intestine, which led to the increased production of SCFAs, activation of the retinol metabolic pathway in PPs, and modulation of inflammatory cytokine or receptor gene expression. These changes increased the proportions of immature DCs and regulatory T cells, leading to immune tolerance.

In this study, *B. bifidum* FN120 was cross-fed with *B. longum* subsp. *longum* FN103 to utilize 2’-FL, resulting in a prolonged time to reach the growth plateau compared with *B. bifidum* FN120 utilizing 2’-FL alone. Previous studies suggested that *B. bifidum* promoted the proliferation of *B. longum* subsp. *longum* via the use of 2’-FL in the intestine, albeit with lower efficiency for its own proliferation.^31^ It was reported that the healthy infant microbiota, dominated by *Bifidobacterium*, showed significantly greater acetic acid production from lactose fermentation than from 2’-FL fermentation, with no difference in propionic and butyric acid production.^32^ In contrast, the gut microbiota of the infants with AD in the present study did not predominantly include *Bifidobacterium*, and the production of acetic and butyric acids during lactose fermentation was significantly greater than that during 2’-FL fermentation. This could be related to the inability of butyrate-producing bacteria, such as *Anaerococcus vaginimassiliensis* and *Clostridium cochlearium*, which were identified in *in vitro* colonic fermentation, to efficiently utilize 2’−FL.

*F. contorta* and *R. lactatiformans* were typical species enriched by 2’-FL and bifidobacteria in the present *in vitro* colonic fermentation study. They were reported to possess α-galactosidase, β-galactosidase, and α-glucosidase activities, with the latter also having α-L-fucosidase activity. These factors contribute to their ability to metabolize polysaccharides to produce acetate, succinate, propionate, butyrate, and valerate.^33,34^ The relative abundance of *B. longum* was significantly increased by 2’-FL and bifidobacterial intervention *in vitro* in the present study, indicating that it obtained monosaccharides from other keystone species, allowing for proliferation. *R. lactatiformans*, owing to its α-glucosidase activity, has the reported potential to degrade 2’-FL and plays a similar role to *B. bifidum* as a cross-feeder.^35^ Additionally, the relative abundance of *Enterococcus faecalis* was increased by both 2’−FL and 2’-FL+Bif supplementation in the present *in vitro* fermentation study, which has the potential to improve AD.^36^ We also found that some low-abundance species detected in the ileum after fermentation broth was transplanted into mice included *A. muciniphila* and *H. faecis*. Another possibility is that these bacteria may be the original intestinal bacteria, and their proliferation is promoted after microbiota transplantation. This is mainly because *H. faecis* is a typical mouse intestinal bacterium. They have been reported to degrade polysaccharides and cross-feed other symbiotic bacteria. *A. muciniphila* has been reported to improve AD,^36^ and *Para. intestinale* could be enriched by *A. muciniphila* administration in mice,^37^ suggesting a potential cross-feeding relationship. The transplantation of the 2’-FL+Bif reshaped microbiota had significantly better AD-protective effects than did the 2’-FL reshaped microbiota alone in the present study. We analyzed the reasons for these effects from various perspectives. First, the ileum is the primary inductive site of immune tolerance via interactions with specific bacteria in the ileum.^28^ The ileal microbiota of the transplanted mice was very different from that of the *in vitro* fermentation system, indicating that the proportion of the transplanted microbiota changed due to the mouse feed and internal environment. Another study showed that the intestinal microbiota of mice treated with antibiotics recovers 2 weeks after microbiota transplantation, but some species are engrafted.^38^ The β diversity levels of the gut microbiota of mice transplanted with different donor microbiota were significantly different, indicating that donor-derived bacteria were the main driving force for the changes in the recipient intestinal microbiota in the present study. There was a negative correlation between the altered bacterial species in the ilea of mice transplanted with 2’-FL+Bif_OXA and the main AD marker, although this correlation did not reach statistical significance. All four SCFAs had significant and strong negative correlations with the AD marker, especially butyrate, in our study. The major upregulated species, *Para. intestinale*, has been reported to possess α-fucosidase and β-galactosidase activities (https://bacdive.dsmz.de/strain/158518) and to efficiently proliferate and produce SCFAs using polysaccharides.^39^ In this regard, the increased abundance of *Para. intestinale* was consistent with higher SCFA concentrations among the mice in the 2’-FL+Bif_OXA group in our study. It has been reported that gut-derived SCFAs can improve epidermal barrier integrity to limit early allergen sensitization.^39^ These findings indicate that SCFAs act as a pivotal nexus within the gut‒skin axis, through which the intestinal microbiota in the 2’-FL+Bif_OXA group of mice exerts their AD-preventive effects.

Second, through transcriptomic analysis, we confirmed that the oral tolerance response in the PPs of 2’-FL+Bif_OXA group mice was induced, resulting in increased proportions of immature DCs and Treg cells within the PPs. Immature DCs, located in the subepithelial dome of PPs, actively survey antigens taken up from the intestinal lumen and play a certain role in immune tolerance induction by promoting Treg differentiation.^40,41^ The retinol metabolic pathway was the primary pathway upregulated in OXA-induced mice in the 2’-FL+Bif_OXA group, which was primarily responsible for converting retinol into retinoic acid (RA) in the present study. RA production promotion was also confirmed by our untargeted metabolomics results. RA can be produced by intestinal epithelial cells or immature DCs and is an essential molecule for promoting Treg differentiation, as reported previously.^42,43^ It has been reported that retinoid transport, synthesis, concentration, and signaling are strongly decreased in the skin of patients with AD.^44^ In the present study, the plasma RA level in the STAB_OXA group was lower than those in the other groups, which is consistent with the results observed in this human study.^44^

The transplantation of microbiota reshaped by 2’-FL+Bif promoted the production of RA in mice in the present study, and there could be two reasons for this phenomenon. First, butyrate has been reported to induce the conversion of vitamin A into RA in CD103^+^ DCs, thereby promoting Treg cell differentiation and facilitating oral tolerance.^45^ Second, the plasma 3-indoleacrylate level was increased in the 2’-FL+Bif_OXA mice in the present study. 3-Indoleacrylate is a gut microbiota-derived tryptophan metabolite that promotes intestinal epithelial barrier function and mitigates inflammatory responses.^46^ Another study revealed that increased intestinal 3-indoleacrylate levels in mice treated with probiotics improved food allergy.^47^ Therefore, 3-indoleacrylate may promote an antiallergic immune response.

In conclusion, our results demonstrated that concurrent supplementation with 2’-FL and specific cross-feeding bifidobacteria promoted the production of SCFAs by the gut microbiota of infants with AD. These findings also provide evidence that this reshaped microbiota promotes the development of anti-AD immune responses by activating the retinol metabolic pathway in mice. These results not only offer novel insights into the therapeutic potential of combining prebiotics and probiotics for managing infant AD but also contribute to the evidence supporting the gut – skin axis as a therapeutic target for allergic diseases. Future clinical trials would be instrumental in translating these insights into practical interventions for managing AD and potentially other immune-mediated conditions.

## Supplementary Material

Supplementary_Materials clean.docx

## Data Availability

The raw sequence data for the 16S rRNA gene are available in the Sequence Read Archive (SRA) under BioProject accessions PRJNA1186070 and PRJNA1185836.
