# Supplementary material for: 2’-FL and cross-feeding bifidobacteria reshaped the gut microbiota of infants with atopic dermatitis ex vivo and prevented dermatitis in mice post-microbiota transplantation through retinol metabolism activation
Source: Gut Microbes. 2025 Mar 2;17(1):2474148. doi: 10.1080/19490976.2025.2474148 (PMC11881859; doi:10.1080/19490976.2025.2474148)
Supplement: Supplementary_Materials clean.docx [file KGMI_A_2474148_SM4714.docx]

**Supplementary Material**

# Supplementary methods

***Genome sequencing and annotation***

*Bifidobacterium bifidum* FN120 and *Bifidobacterium longum* subsp. *longum* FN103 were cultivated in De Man, Rogosa, and Sharpe broth for an incubation period of 18 hours at 37°C under anaerobic conditions. The bacterial cells were harvested via centrifugation at 12,000 × g for 5 minutes, following which genomic DNA was extracted from the cell pellets using a Genomic DNA Purification Kit (Thermo Scientific, USA), according to the manufacturer's guidelines. The quantity and purity of the DNA were assessed spectrophotometrically by measuring absorbance at 260 nm employing a NanoDrop ND-1000 UV-Vis Spectrophotometer (Thermo Scientific, USA). Sequencing of the complete genomes was performed using the PacBio Sequel II and Illumina NovaSeq PE150 platforms, as described.^1^ Functional annotations were conducted using EggNOGmapper v2 (version 2.0) for protein clustering into orthologous groups, and using the run_dbcan python package v. 4.0.0 for the identification of genes related to human milk oligosaccharide utilization. A phylogenomic tree of each strain with other strain form NCBI genome database was reconstructed utilizing the UBCG tool (www.ezbiocloud.net/tools/ubcg), which employs 92 single-copy protein-coding sequences for its analysis.

***Peyer’s patches (PPs) collection and RNA sequencing***

The small intestine was transected approximately 1 cm proximal to the cecum, followed by the excision of adipose tissue, mesenteric lymph nodes, and surrounding connective tissues. PPs were extracted by applying tension to the patch with curved forceps and subsequent excision with scissors, taking care to minimize damage to the adjacent intestinal wall. The PPs presented as minute protruding nodules with a whitish-grey hue (1-2 mm in diameter), analogous to lymph nodes were embedded within the yellow-brown outer layer of the intestinal wall. Typically, 5 to 10 PPs were harvested from each mouse (4 mice per group). Total RNA was extracted from pooled PPs of the small intestine. A quantity of 1 μg of total RNA was diluted in 25 μL of nuclease-free water. RNA sequencing libraries were assembled with the NEBNext Ultra kit for Illumina, adhering to the protocol provided by New England Biolabs. Subsequently, paired-end sequencing was performed on the NovaSeq 6000 by Biomarker Technologies, China.

***Procedure of*** ***ultra-high performance liquid chromatography-quadrupole-time of flight***

A 5 μL sample was injected on a Waters Acquity ultra-high performance liquid chromatography BEH Amide column (150 mm length × 2.1 mm id; 1.7 μm particle size) maintained at 45°C. A Waters Acquity VanGuard BEH Amide pre-column (5 mm × 2.1 mm id; 1.7 μm particle size) was used as a guard column. Mobile phase A was 100% LC-MS grade water with 10 mM ammonium formate and 0.125% formic acid and mobile phase B was 95:5 v/v acetonitrile: water with 10 mM ammonium formate and 0.125% formic acid. Gradient was started at 100% (B) for 2 min, 70% (B) at 7.7 min, 40% (B) at 9.5 min, 30% (B) at 10.25 min, 100% (B) at 12.75 min and isocratic until 16.75 min. The column flow was 0.4 mL/min. Agilent 1290 infinity UPLC instrument was used. The MS data were acquired using an Agilent 6530B Quadrupole Time-of-Flight mass spectrometer equipped with an ion funnel (iFunnel) electro spray ionization source. Data were acquired in ESI (+) modes. Source parameters were gas temperature 325°C, drying gas flow 8 L/min, nebulizer 35 psig, sheath gas temp 350°C, sheath gas flow 11 L/min and Fragmentor 175 V. Mass scan range was 50-1700 and acquisition rate was 2 spectra per second. Reference masses 121.050 and 922.009 were used for continuous calibration of the mass accuracy. Data were acquired in the centroid mode. Raw data files (.d) format were converted to .abf using the ABF converter (https://www.reifycs.com/AbfConverter/index.html). MS-DIAL software was used for peak detection, alignment, gap filling and annotations. Adducts were grouped and duplicate peaks were removed using the MS-FLO software.

***Bioinformatics analysis***

The CIBERSORT algorithm was employed to estimate the proportions of immune cell subtypes in Peyer's patches from RNA sequencing data. The R package ClusterProfiler was utilized for Gene Set Enrichment Analysis (GSEA), and the "Pathview" R package was used to visualize KEGG pathways of interest.

For microbiota analysis, the *unoise* function of USEARCH was used to generate amplicon sequence variants (ASVs) and representative sequences. The *usearch_global* function of VSEARCH was employed to create the feature table. Species annotation was performed using BLCA^2^ with the RefSeq database from June 2024. Alpha diversity was assessed using the vegan package, and the Permanova function from the micro4all package was used to select the optimal distance calculation method. The *Betadispersion* function was applied to analyze the significance of dispersion between groups. Beta diversity was calculated using the pairwise.adoni (PERMANOVA) function from the "pairwiseAdonis" R package with BH correction for principal co-ordinates analysis (PCoA), and the *anosim* (ANOSIM) function for nonmetric multidimensional scaling (NMDS). Differential species analysis between groups was conducted using the STAMP software.

MS-DIAL software (version 4.70) was utilized for spectral deconvolution, peak alignment, gap filling, and peak identification. The .mgf files, containing MS1 and MS2 data, were employed to retrieve metabolite details at both MS1 and MS2 levels using the in-house MS2Compound tool.^3^ Metabolites from the KEGG database, which excludes food compounds, environmental pollutants, and drug molecules, were computationally fragmented using metabolite SMILES IDs as inputs in the Competitive Fragmentation Modeling-ID (CFM-ID) tool.^4^ These fragmented details served as the theoretical database for plasma metabolite searches. Parameters such as a precursor tolerance of 0.05 Da, a fragment tolerance of 0.5 Da, and a minimum of two fragment matches were established for metabolite searches against the simplified KEGG database. The selection criteria for compounds were based on a rank of 1 and the highest mass spectrometry (MS) score. Additionally, m/z features without metabolite assignment at the MS2 level were assigned at the MS1 level. The Integrated mass spectrometry-based untargeted metabolomics data mining (IP4M) software was employed for further analysis, encompassing peak table pre-processing and orthogonal partial least squares discriminant analysis (OPLS-DA). Differential metabolites in metabolic profiles between groups were identified based on a variable importance in the projection (VIP) threshold of 1 from the OPLS-DA model.

***Reference***

1. Zhang Y, Zhou Q, Chen Z, Ye Z, Jin Y, Li P, & Gu Q. Probiotic characteristics and whole genome sequence analysis of *Lactiplantibacillus plantarum* ZFM518 isolated from infant feces. *Food Frontiers.* 2024; *5*(5), 2235-2248. doi.org/10.1002/fft2.444.
2. Gao X, Lin H, Revanna K, Dong Q. A Bayesian taxonomic classification method for 16S rRNA gene sequences with improved species-level accuracy. BMC Bioinformatics. 2017; 18:247. doi:10.1186/s12859-017-1670-4.
3. Behera SK, Kasaragod S, Karthikkeyan G, Narayana Kotimoole C, Raju R, Prasad TSK, Subbannayya Y. MS2Compound: A User-friendly compound identification tool for LC-MS/MS-based metabolomics data. OMICS 2021; 25:389–99. doi: 10.1089/omi.2021.0051.
4. Wang F, Liigand J, Tian S, Arndt D, Greiner R, Wishart DS. CFM-ID 4.0: More accurate ESI-MS/MS spectral prediction and compound identification. Anal Chem. 2021; 93:11692–700. doi:10.1021/acs.analchem.1c01465.

# Supplementary Table

Table S1 Population baseline information.

| Item | donor1 | donor2 | donor3 | donor4 | donor5 | donor6 |
| --- | --- | --- | --- | --- | --- | --- |
| age (days) | 32 | 42 | 22 | 45 | 42 | 42 |
| Birth weight | 3.1 | 2.48 | 2.84 | 3 | 3.25 | 2.48 |
| Pre-pregnancy weight (kg) | 65 | 63 | 53 | 75 | 54 | 60 |
| Prenatal weight (kg) | 77 | 72.5 | 61.7 | 84 | 67.5 | 75 |
| Mother's age | 26 | 27 | 32 | 24 | 27 | 24 |
| Mother's height (m) | 1.6 | 1.7 | 1.63 | 1.69 | 1.56 | 1.65 |
| Weight gain during pregnancy | 12 | 9.5 | 8.7 | 9 | 13.5 | 15 |
| Prenatal BMI (kg/m^2^) | 23.59 | 21.8 | 19.95 | 26.26 | 22.19 | 22.04 |
| Postpartum BMI (kg/m^2^) | 30.08 | 25.09 | 23.22 | 29.41 | 27.74 | 27.55 |
| Antibiotic use* | no | no | no | no | no | no |
| Probiotics consumption* | no | no | no | no | no | no |
| Mode of delivery | VD | CS | VD | CS | VD | CS |

* One month of antibiotic and probiotics consumption preceding sampling; BMI, body mass index; CS, cesarean section; VD, Vaginal delivery

Table S2 The composition of the mZMB media (g/L).

| Composition | | Final concentration |
| --- | --- | --- |
| Part I  Phosphate buffer system | KH_2_PO_4_ | 6.288 |
|  | K_2_HPO_4_ | 12.852 |
|  | (NH_4_)_2_SO_4_ | 2 |
|  | NaCl | 6 |
| Macro organic matter | Tryptone | 64.8 |
|  | Lactose or 2'-fucosyllactose | 10 |
|  | Pig Bile Salts | 0.1 |
|  | Cysteine ​​hydrochloride | 2 |
|  | Mucin from porcine stomach Type II. | 8 |
| Part II  Vitamins | Myo-inositol | 0.002 |
|  | Calcium pantothenate | 0.0012 |
|  | Niacin | 0.0009 |
|  | Pyridoxal HCL | 0.0048 |
|  | Folic Acid | 0.0005595 |
|  | p-Aminobenzoic acid | 0.00005595 |
|  | Biotin | 0.006 |
|  | Thiamine | 0.0005595 |
|  | Riboflavin | 0.0009 |
| Antioxidants | Glutathione | 0.015 |
|  | Thioctic acid | 0.001 |
|  | Hemin | 0.01 |
| Short-chain fatty acids | Potassium acetate | 0.901 |
| Nucleic acid | Adenine | 0.01101 |
|  | Guanine | 0.005505 |
|  | Uracil | 0.02202 |
|  | Xanthine | 0.00367 |
| Part III  Minerals | MgSO_4_•7H_2_O | 1 |
|  | FeSO_4_•7H_2_O | 0.004 |
|  | ZnSO_4_•7H_2_O | 0.005 |
|  | (NH_4_)_6_Mo_7_O_2_•4H_2_O | 0.00019 |
|  | MnSO_4_•4H_2_O | 0.00038 |
|  | CaSO_4_•4H_2_O | 0.038 |
|  | CoSO_4_•6H_2_O | 0.000228 |
|  | CuSO_4_•5H_2_O | 0.00019 |
|  | H_3_BO_3_ | 0.00076 |
|  | K_2_SO_4_ | 0.0228 |
|  | KI | 0.000114 |
| Part IV  Chelating agents | EDTA | 0.0075 |
|  | Nitriliotriacetic Acid | 0.0075 |

Table S3. Human milk oligosaccharide utilization related genes of FN120 and FN103

| dbCAN subfam | Enzym name | Target | Gene ID | E Value | Strain |
| --- | --- | --- | --- | --- | --- |
| GH33_e129 | 2,3-2,6-a-sialidase | Neu5Acα2-3Gal/Neu5Acα2-6Gal/Neu5Acα2-6GlcNAc | Contigs_1_376756_209 | 7.1E-144 | FN120 |
| GH33_e129 | 2,3-2,6-a-sialidase | Neu5Acα2-3Gal/Neu5Acα2-6Gal/Neu5Acα2-6GlcNAc | Contigs_1_376756_210 | 3.7E-141 | FN120 |
| GH112_e0 | GNB/LNB phosphorylase | Galβ1-3GlcNAc | Contigs_3_350980_175 | 0 | FN103 |
| GH112_e0 | GNB/LNB phosphorylase | Galβ1-3GlcNAc | Contigs_11_88762_79 | 8.4E-246 | FN120 |
| GH112_e0 | GNB/LNB phosphorylase | Galβ1-3GlcNAc | Contigs_9_112297_54 | 0 | FN120 |
| GH20_e24 | lacto-N-biosidase | GlcNAcβ1-3Gal/GlcNAcβ1-6Gal | Contigs_7_75643_56 | 0 | FN103 |
| GH20_e7 | lacto-N-biosidase | GlcNAcβ1-3Gal/GlcNAcβ1-6Gal | Contigs_11_88762_41 | 1.9E-273 | FN120 |
| GH20_e1 | lacto-N-biosidase | GlcNAcβ1-3Gal/GlcNAcβ1-6Gal | Contigs_1_376756_142 | 2.6E-167 | FN120 |
| GH20_e14 | lacto-N-biosidase | GlcNAcβ1-3Gal/GlcNAcβ1-6Gal | Contigs_3_316280_173 | 6.7E-257 | FN120 |
| GH20_e20 | lacto-N-biosidase | GlcNAcβ1-3Gal/GlcNAcβ1-6Gal | Contigs_3_316280_81 | 1.9E-174 | FN120 |
| GH136_e10 | lacto-N-biosidase | GlcNAcβ1-3Gal | Contigs_4_289504_4 | 2.8E-272 | FN120 |
| GH95_e0 | α-1,2-L-fucosidase | Fucα1-2Gal | Contigs_9_112297_10 | 3.4E-240 | FN120 |
| GH29_e78 | α-L-fucosidase | Fucα1-3Gal/Fucα1-4Gal/Fucα1-3GlcNAc/Fucα1-4GlcNAc | Contigs_3_316280_14 | 7.9E-262 | FN120 |
| GH29_e30 | α-L-fucosidase | Fucα1-3Gal/Fucα1-4Gal/Fucα1-3GlcNAc/Fucα1-4GlcNAc | Contigs_9_112297_52 | 2.4E-140 | FN120 |

The green-shaded enzyme is the key enzyme responsible for the degradation of 2'-fucosyllactose. dbCAN, Carbohydrate-active enzymes database; Gal, galactose; GlcNAc, N-Acetyl-D-glucosamine; GNB, galacto-*N*-biose; FN120, *B. bifidum* FN120; FN103, *B. longum* subsp. *longum* FN103; LNB, Lacto-N-Biosidase; Neu5Ac, N-acetylneuraminic acid.

Table S4 Human milk oligosaccharide transport related genes of FN120 and FN103

| Gene_id | COG:Identity | COG:E_value | InterPro_  description | Strain |
| --- | --- | --- | --- | --- |
| Contigs_1_1004974_62 | 83.6 | 1.10E-144 | MetI | FN120 |
| Contigs_2_354061_136 | 61.3 | 7.70E-101 | MetI | FN120 |
| Contigs_3_350980_177 | 84.5 | 1.20E-152 | MetI | FN120 |
| Contigs_5_109756_21 | 45.4 | 5.70E-67 | MetI | FN120 |
| Contigs_8_50761_41 | 84.9 | 1.20E-144 | MetI | FN120 |
| Contigs_1_1004974_63 | 82.1 | 4.40E-150 | MetI | FN120 |
| Contigs_2_354061_137 | 58.1 | 2.40E-90 | MetI | FN120 |
| Contigs_3_350980_176 | 84.5 | 3.90E-148 | MetI | FN120 |
| Contigs_5_109756_20 | 52.6 | 2.30E-84 | MetI | FN120 |
| Contigs_8_50761_40 | 80.5 | 2.30E-151 | MetI | FN120 |
| Contigs_2_356640_2 | 91.1 | 5.60E-163 | MetI | FN103 |
| Contigs_2_356640_3 | 89.8 | 1.60E-165 | MetI | FN103 |
| Contigs_6_140164_110 | 58.6 | 4.20E-90 | MetI | FN103 |
| Contigs_9_112297_49 | 73.6 | 8.50E-124 | MetI | FN103 |
| Contigs_9_112297_50 | 81.7 | 1.30E-140 | MetI | FN103 |
| Contigs_1_376756_180 | 95.5 | 1.90E-202 | MalK | FN103 |
| Contigs_1_376756_88 | 64.7 | 3.10E-145 | MalK | FN103 |
| Contigs_2_356640_69 | 68.4 | 3.50E-94 | MalK | FN103 |
| Contigs_10_40542_23 | 40.5 | 1.20E-86 | SBPs | FN120 |
| Contigs_9_112297_48 | 80.4 | 8.30E-213 | SBPs | FN103 |

COG, clusters of orthologous groups of proteins; FN103, *B. longum* subsp. *longum* FN103; FN120, *B. bifidum* FN120; MalK; ABC transporter-like, ATP-binding domain; MetI, ABC transporter type 1, transmembrane domain MetI-like; SBPs, Bacterial extracellular solute-binding protein.

Table S5. Permanova alysis based on different distance method for microbiota of continuous fermentation

|  | Df | Sum.Sq | R^2^ | F | Pr ( > F) | Distances |
| --- | --- | --- | --- | --- | --- | --- |
| Model | 2 | 1.21 | 0.51 | 7.92 | 0.001 | bray |
| Residual | 15 | 1.15 | 0.49 |  |  |  |
| Total | 17 | 2.36 | 1 |  |  |  |
| Model1 | 2 | 0.04 | 0.13 | 1.07 | 0.324 | unifrac |
| Residual1 | 15 | 0.25 | 0.87 |  |  |  |
| Total1 | 17 | 0.293 | 1 |  |  |  |
| Model2 | 2 | 0.54 | 0.42 | 5.43 | 0.001 | wunifrac |
| Residual2 | 15 | 0.75 | 0.58 |  |  |  |
| Total2 | 17 | 1.30 | 1 |  |  |  |

Bray-Curtis distance explain the most variance expressed as **R^2^**.

Table S6. Multiple betadisper test with three distances measures for microbiota of continuous fermentation.

|  | Df | Sum.Sq | Mean.Sq | F | N.Perm | Pr..F. | Distances |
| --- | --- | --- | --- | --- | --- | --- | --- |
| Groups | 2 | 0.04 | 0.02 | 1.10 | 999 | 0.372 | bray |
| Residuals | 15 | 0.27 | 0.02 |  |  |  | bray |
| Groups1 | 2 | 0.00 | 0.00 | 0.13 | 999 | 0.891 | unifrac |
| Residuals1 | 15 | 0.07 | 0.00 |  |  |  | unifrac |
| Groups2 | 2 | 0.04 | 0.02 | 1.02 | 999 | 0.378 | wunifrac |
| Residuals2 | 15 | 0.26 | 0.02 |  |  |  | wunifrac |

Dispersion was not statistically significantly different between the three groups.

Table S7. Permanova analysis based on different distance method for microbiota of mice ileum

|  | Df | SumOfSqs | R2 | F | Pr ( > F ) | Distances |
| --- | --- | --- | --- | --- | --- | --- |
| Model | 5 | 2.41 | 0.29 | 1.98 | 0.001 | bray |
| Residual | 24 | 5.84 | 0.71 |  |  | bray |
| Total | 29 | 8.25 | 1 |  |  | bray |
| Model1 | 5 | 0.63 | 0.24 | 1.48 | 0.001 | unifrac |
| Residual1 | 24 | 2.053 | 0.76 |  |  | unifrac |
| Total1 | 29 | 2.68 | 1 |  |  | unifrac |
| Model2 | 5 | 0.92 | 0.21 | 1.29 | 0.093 | wunifrac |
| Residual2 | 24 | 3.42 | 0.79 |  |  | wunifrac |
| Total2 | 29 | 4.34 | 1 |  |  | wunifrac |

Bray-Curtis distance explain the most variance (expressed as **R^2^**).

Table S8. Multiple betadisper test with three distances measures for microbiota of FMT mice experiment

|  | Df | Sum.Sq | Mean.Sq | F | N.Perm | Pr. F. | Distances |
| --- | --- | --- | --- | --- | --- | --- | --- |
| Groups | 5 | 0.08 | 0.02 | 1.12 | 999 | 0.372 | bray |
| Residuals | 24 | 0.35 | 0.01 |  |  |  | bray |
| Groups1 | 5 | 0.01 | 0.00 | 0.59 | 999 | 0.706 | unifrac |
| Residuals1 | 24 | 0.08 | 0.00 |  |  |  | unifrac |
| Groups2 | 5 | 0.10 | 0.02 | 0.92 | 999 | 0.483 | wunifrac |
| Residuals2 | 24 | 0.50 | 0.02 |  |  |  | wunifrac |

Dispersion was not statistically significantly different between the three groups, the PERMANOVA results can be sure.

# Supplementary Figures

Figure S1


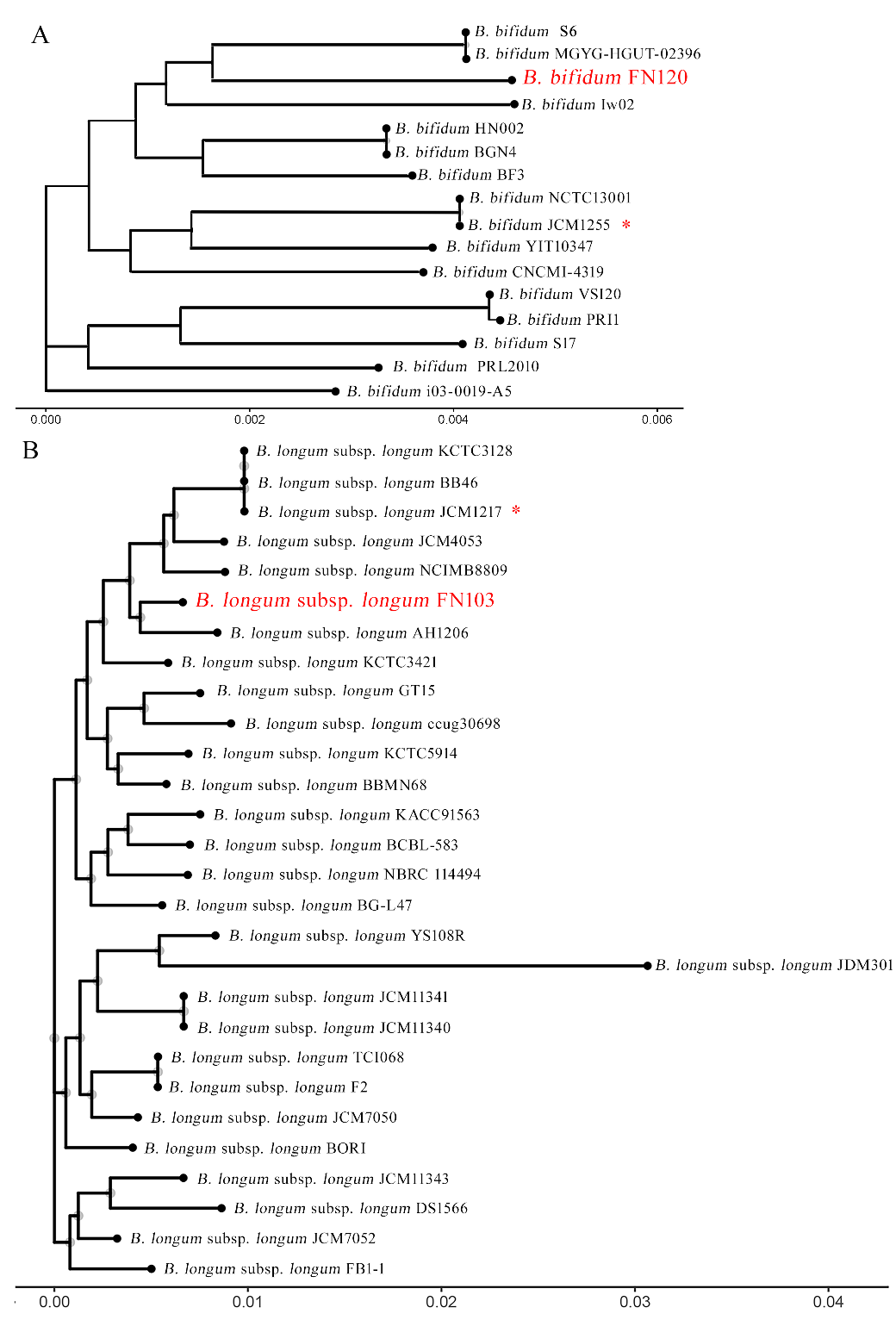


Figure S1**.** Phylogenetic tree of 16 *Bifidobacterium bifidum* and 28 *Bifidobacterium longum* subspecies. *long* strains with complete genome from NCBI database using the concatenated sequences of 92 core single-copy genes via the UBCG pipeline. Node labels indicate the Gene Support Index value which represents the number of genes (from a total of 92) whose common sequences group the taxa together within a branch. Type strains were shown with a red asterisk.

Figure S2


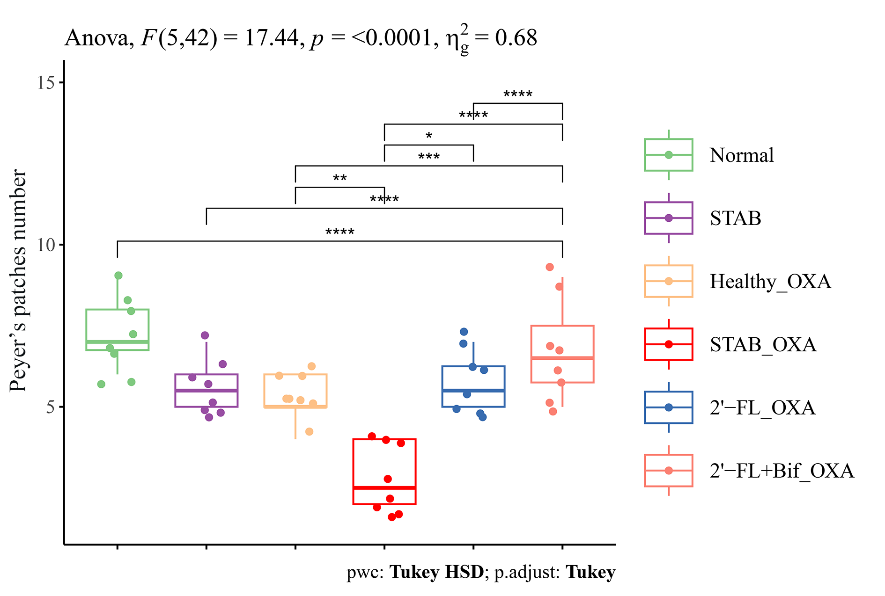


Figure S2. Effects of transplantation of *in vitro* fermented faecal microbiota of infants with atopic dermatitis supplemented with 2'-FL or 2'-FL and Bif on Peyer’s patches number of oxazolone-sensitized BALB/c mice. **p* < 0.05, ***p* < 0.01, ****p* < 0.001, according to one-way analysis of variance (ANOVA), followed by individual comparisons with Tukey–Kramer's post-hoc test; 2’-FL_OXA, 2’-FL+Bif_OXA, STAB_OXA, Healthy_OXA, transplantation of in vitro microbiota supplemented with 2'-FL, 2'-FL+Bif, that at STAB stage, and of fecal microbiota from healthy infants, respectively, and induction of AD with OXA; AD, atopic dermatitis; Bif, *Bifidobacterium* *longum* subsp. *longum* FN103 and *Bifidobacterium* *bifidum* FN120 capable of cross-feeding on 2’-FL; Normal, Healthy mouse control; OXA, oxazolone; STAB, mice transplanted with stabilization stage faecal microbiota of donor 2 without AD induction.

Figure S3


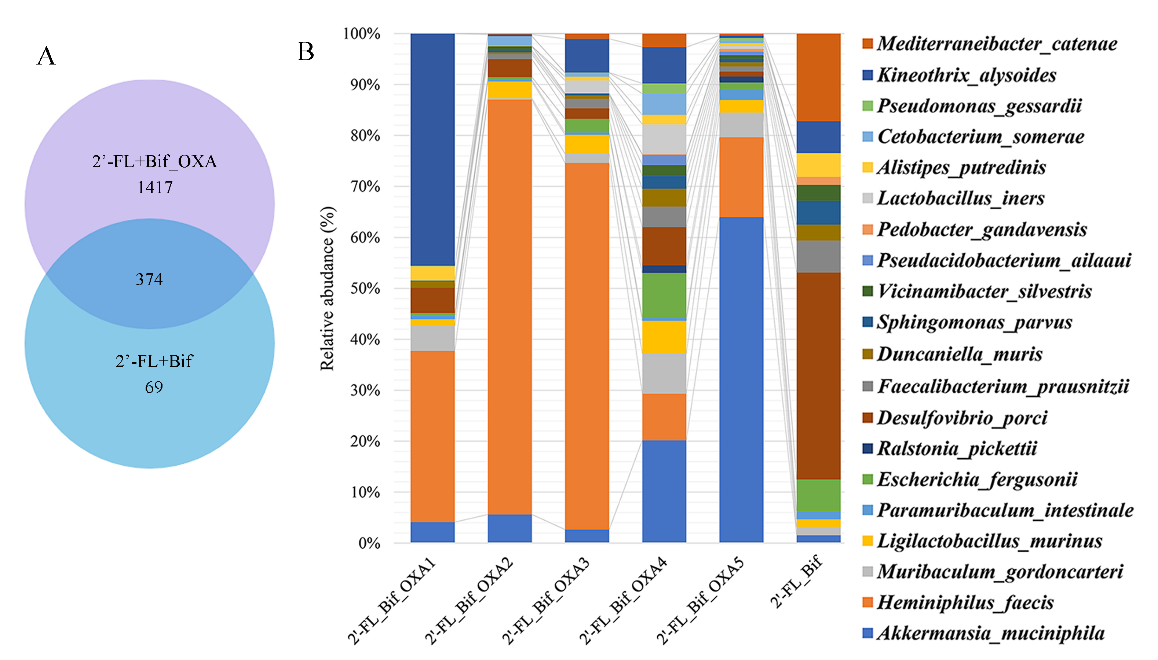


Figure S3. Shared amplicon sequence variants (347) in the microbiota from the 2'-FL+Bif phase of *in vitro* fermentation of donor 2 and ileal microbiota of the 2'-FL+Bif_OXA group (A), and (B) the corresponding top 20 species profile. 2’-FL+Bif_OXA transplantation of *in vitro* microbiota supplemented with 2'-FL+Bif. 2 ′-FL, 2′-Fucosyllactose; Bif, mixture of *Bifidobacterium longum* subsp. *longum* FN103 and *Bifidobacterium* *bifidum* FN120 capable of cross-feeding on 2’-FL.
